# Network Pharmacology as a Tool to Investigate the Antioxidant and Anti-Inflammatory Potential of Plant Secondary Metabolites—A Review and Perspectives

**DOI:** 10.3390/ijms26146678

**Published:** 2025-07-11

**Authors:** Anna Merecz-Sadowska, Arkadiusz Sadowski, Hanna Zielińska-Bliźniewska, Karolina Zajdel, Radosław Zajdel

**Affiliations:** 1 Department of Economic and Medical Informatics, University of Lodz, 90-214 Lodz, Poland; arkadiusz.sadowski@uni.lodz.pl; 2 Department of Allergology and Respiratory Rehabilitation, Medical University of Lodz, 90-725 Lodz, Poland; hanna.zielinska-blizniewska@umed.lodz.pl; 3 Department of Medical Informatics and Statistics, Medical University of Lodz, 90-645 Lodz, Poland; karolina.smigiel@umed.lodz.pl

**Keywords:** network pharmacology, plant secondary metabolites, antioxidant, anti-inflammatory, systems pharmacology, natural products, molecular targets, pathway analysis

## Abstract

Plant secondary metabolites possess significant antioxidant and anti-inflammatory properties, but their complex polypharmacological mechanisms remain poorly understood. Network pharmacology has emerged as a powerful systems-level approach for investigating multi-target interactions of natural products. This review systematically analyzes network pharmacology applications in elucidating the antioxidant and anti-inflammatory mechanisms of plant metabolites, evaluating concordance between computational predictions and experimental validation. A comprehensive literature search was conducted across major databases (2015–2025), focusing on network pharmacology studies with experimental validation. Analysis revealed remarkable convergence toward common molecular mechanisms, despite diverse chemical structures. For antioxidant activities, the Nrf2/KEAP1/ARE pathway emerged as the most frequently validated mechanism, along with PI3K/AKT, MAPK, and NF-κB signaling. Anti-inflammatory mechanisms consistently involved NF-κB, MAPK, and PI3K/AKT pathways. Key targets, including AKT1, TNF-α, COX-2, NFKB1, and RELA, were repeatedly identified. Flavonoids, phenolic acids, and terpenoids dominated as bioactive compounds. Molecular docking studies supported predicted interactions, with experimental validation showing good concordance for pathway modulation and cytokine regulation. Network pharmacology provides a valuable framework for investigating the complex bioactivities of plant metabolites. The convergence toward common regulatory hubs suggests that natural compounds achieve protective effects by modulating central nodes that integrate redox balance and inflammatory responses. Despite limitations, including database dependency, integrating network pharmacology with experimental validation accelerates mechanistic understanding in natural-product drug discovery.

## 1. Introduction

For millennia, plants have served as a fundamental source of bioactive compounds with diverse beneficial properties, largely due to their capacity to synthesize a wide array of secondary metabolites. These organic molecules differ from primary metabolites by fulfilling primarily ecological functions, including defense against herbivores and pathogens, protection against abiotic stresses, and mediation of interactions with pollinators. The evolutionary pressures driving these functions have generated remarkable chemical diversity among secondary metabolites, establishing them as an exceptionally rich source of bioactive compounds that exhibit properties including antioxidant and anti-inflammatory activities [1,2,3].

Oxidative stress and inflammation are intricately linked pathophysiological processes underlying numerous chronic diseases. Oxidative stress occurs when the balance between pro-oxidant generation and antioxidant defense is disrupted, resulting in an accumulation of reactive oxygen species (ROS) and reactive nitrogen species (RNS) that damage cellular components such as lipids, proteins, and DNA. Cellular antioxidant defenses include enzymatic components like superoxide dismutase (SOD), catalase (CAT), and glutathione peroxidases (GPx), which are regulated by key transcription factors, most notably Nuclear factor erythroid 2-related factor 2 (Nrf2). Oxidative stress constitutes a major etiological factor in cardiovascular diseases, metabolic disorders, malignancies, and neurodegenerative conditions. Moreover, while inflammation represents a critical protective response, its chronic dysregulation drives global morbidity and mortality [4,5]. Inflammatory processes are governed by pivotal signaling pathways, primarily the Nuclear Factor kappa-light-chain-enhancer of activated B cells (NF-κB), which controls the transcription of pro-inflammatory genes encoding cytokines like Tumor Necrosis Factor-alpha (TNF-α), Interleukin-1beta (IL-1β), and Interleukin-6 (IL-6), as well as enzymes such as Cyclooxygenase-2 (COX-2) and Inducible Nitric Oxide Synthase (iNOS). The bidirectional relationship between these processes—where ROS can activate pro-inflammatory pathways like NF-κB, and activated inflammatory cells, in turn, produce substantial quantities of ROS—creates a vicious cycle. Consequently, compounds possessing both anti-inflammatory and antioxidant properties are crucial for interrupting this cycle and maintaining tissue homeostasis [6,7,8].

Despite the immense potential of plant secondary metabolites, their identification, isolation, and characterization remain labor-intensive and costly processes that typically yield limited quantities of structurally complex compounds [9,10]. In response, computational (in silico) methods have undergone substantial advancement, becoming integral to research on natural products [11]. These techniques—encompassing molecular modeling, chemoinformatics, bioinformatics, artificial intelligence (AI), and machine learning (ML)—enable the prediction of pharmacological activities, modeling of molecular interactions, evaluation of pharmacokinetic properties, and analysis of large datasets. This evolution represents a paradigm shift toward systematic, data-driven strategies that complement traditional approaches by facilitating hypothesis generation and prioritization of promising candidates [12,13,14].

Network pharmacology (NP) has emerged as a particularly powerful computational approach for investigating the complex bioactivities of natural products. Unlike traditional single-target paradigms, NP embraces the polypharmacological nature of plant extracts by analyzing interactions within biological networks. The analytical pipeline typically involves the following steps: (1) identification of compounds from natural sources; (2) target prediction using computational tools; (3) compilation of disease-associated genes; (4) network construction, integrating compounds, targets, and disease-related proteins; (5) topological analysis to identify key nodes; (6) pathway enrichment analysis; and (7) in silico validation through molecular docking [15,16,17,18].

This review provides a comprehensive analysis of NP applications for elucidating the antioxidant and anti-inflammatory properties of plant secondary metabolites. We systematically examine studies employing NP approaches to investigate the mechanisms by which these compounds modulate oxidative stress and inflammation, with particular emphasis on comparing computational predictions with in vitro validation results. By mapping advances, challenges, and emerging trends at this intersection, we identify current limitations and promising directions for future computational and experimental investigations in research on natural products.

## 2. Review Methodology

To provide a comprehensive overview of NP applications in investigating the antioxidant and anti-inflammatory potential of plant secondary metabolites, a systematic search of the literature was conducted. The objective was to identify and synthesize original research articles employing NP methodologies to elucidate the mechanisms underlying these specific biological activities of plant-derived compounds.

The literature search encompassed major scientific databases, including PubMed, Scopus, Web of Science, and Google Scholar, ensuring broad coverage of relevant publications. The search strategy utilized combinations of keywords related to the core concepts: “network pharmacology,” “systems pharmacology,” “in silico,” AND “plant,” “medicinal plant,” “herb,” “natural product,” “phytochemical,” “secondary metabolite,” AND “antioxidant,” “oxidative stress,” “Nrf2,” “ARE,” “redox,” AND/OR “anti-inflammatory,” “inflammation,” “cytokine,” “NF-κB,” “MAPK,” “COX-2,” “iNOS,” “TNF,” “interleukin.” Boolean operators were employed to optimize search specificity and sensitivity. The inclusion of specific molecular targets and pathways (e.g., “Nrf2,” “COX-2”) alongside broader terms was a deliberate strategy employed to enhance search sensitivity, ensuring the capture of mechanistically focused studies that might not explicitly use the more general keywords in their titles or abstracts. The search primarily focused on the literature published from January 2015 up until May 2025.

The inclusion criteria were as follows: (1) original research papers published in peer-reviewed journals in English; (2) studies applying NP approaches (including target prediction, network construction, and pathway analysis) to investigate antioxidant and/or anti-inflammatory mechanisms of plant secondary metabolites, extracts, or formulations; and (3) studies integrating in silico findings with experimental validation (in vitro or in vivo) or molecular docking comparisons.

The following exclusion criteria were included: (1) review articles, meta-analyses, conference abstracts, book chapters, and editorials; (2) studies employing in silico methods other than NP as the primary approach; (3) research focusing primarily on biological activities other than antioxidant or anti-inflammatory properties; (4) studies investigating metabolites from non-plant sources; and (5) non-English publications.

**Figure 1:**
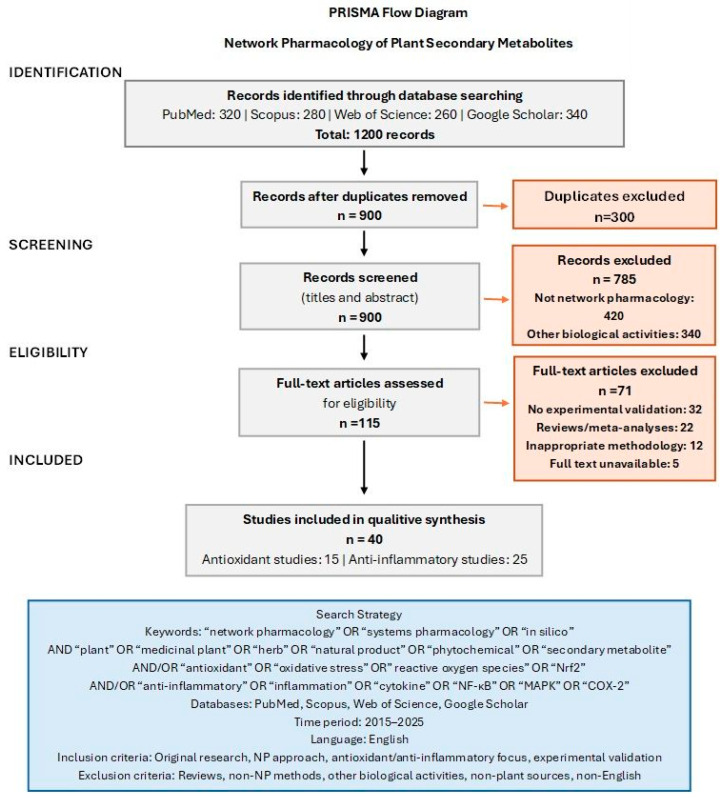
PRISMA flow diagram.

The selection process involved initial screening of titles and abstracts for relevance, with the removal of duplicates across databases. The full texts of potentially relevant articles were subsequently assessed for eligibility. Articles meeting all the inclusion criteria and none of the exclusion criteria were selected for detailed analysis (Figure 1). The studies summarized in Supplementary Tables S1 and S2 represent the core literature identified through this systematic approach.

## 3. Typical Workflow of NP in Plant Secondary Metabolite Research

NP has emerged as a powerful systems-level approach for unraveling the complex mechanisms of action of natural products [17,19]. Plant extracts contain numerous secondary metabolites that often exert therapeutic effects through polypharmacology—interactions with multiple molecular targets, rather than a single one [20,21]. NP integrates methodologies from systems biology, bioinformatics, cheminformatics, and pharmacology to systematically investigate these multi-component, multi-target interactions within biological networks [22,23].

The typical NP workflow follows a structured pipeline from compound identification to mechanistic hypothesis generation [24,25]. First, compound identification involves either retrieving known compounds from databases such as the Traditional Chinese Medicine Systems Pharmacology Database and Analysis Platform (TCMSP) [26], PubChem [27], ChemSpider [28], or the Encyclopedia of Traditional Chinese Medicine (ETCM) [29], or characterizing uncharacterized extracts using analytical techniques like Ultra-Performance Liquid Chromatography–Tandem Mass Spectrometry (UPLC-MS/MS) or Gas Chromatography–Mass Spectrometry (GC-MS) [30,31]. Given the large number of compounds, filtering based on Absorption, Distribution, Metabolism, and Excretion (ADME) properties (Oral Bioavailability, Drug-Likeness, Lipinski’s Rule of Five) [32] using platforms like SwissADME [33] or TCMSP [26] prioritizes compounds that are likely to exert biological effects.

Target prediction is a critical step that employs various computational strategies to identify the potential molecular targets of bioactive compounds. The most common strategies are ligand-based approaches, which leverage the principle that structurally similar molecules often bind to similar protein targets. Prominent tools employing this strategy include SwissTargetPrediction [34], which uses a combination of 2D and 3D similarity; the Similarity Ensemble Approach (SEA) [35]; and PharmMapper [36], which utilizes pharmacophore mapping. As an alternative, known compound–target interaction data can be retrieved directly from comprehensive databases such as the Search Tool for Interactions of Chemicals (STITCH) [37] and ChEMBL [38]. Following prediction or retrieval, the identified targets are standardized using universal identifiers, typically from the Universal Protein Resource (UniProt) database [39]. Concurrently, disease-associated genes are compiled from databases such as GeneCards [40], Online Mendelian Inheritance in Man (OMIM) [41,42], Disease Gene Network (DisGeNET) [42], Comparative Toxicogenomics Database (CTD) [43], or Kyoto Encyclopedia of Genes and Genomes—DISEASE (KEGG DISEASE) [44]. This dual strategy, which integrates ligand-based predictions with disease-associated gene data, is a fundamental strength of the NP methodology, allowing researchers to pinpoint the most relevant intersections between a compound’s potential bioactivity and the molecular basis of a disease.

The core of NP involves network construction and analysis. Compound–target networks visualize interactions between bioactive compounds and predicted targets. Protein–Protein Interaction (PPI) networks, constructed using Search Tool for the Retrieval of Interacting Genes/Proteins (STRING) [45] or Biological General Repository for Interaction Datasets (BioGRID) [46] data, reveal functional relationships among targets. Cytoscape (version 3.10.3) [47] enables network visualization and topological analysis using plugins like CytoHubba (version 0.1) [48] to identify hub genes based on degree centrality or betweenness centrality.

Functional enrichment analysis elucidates biological significance through Gene Ontology (GO) analysis [49] and KEGG pathway analysis [44,50], using tools like Database for Annotation, Visualization and Integrated Discovery (DAVID) (https://david.ncifcrf.gov, accessed on 15 May 2025) [51] or Metascape (https://metascape.org, accessed on 15 May 2025) [52]. In antioxidant/anti-inflammatory research, commonly enriched pathways include Nrf2 signaling, Mitogen-Activated Protein Kinase (MAPK) signaling, Phosphatidylinositol 3-kinase—Protein Kinase B (PI3K-AKT) signaling, NF-κB signaling, and Hypoxia-Inducible Factor 1 (HIF-1) signaling.

Finally, molecular docking simulations using software like AutoDock Vina (version 1.1.2) [53] or MOE (MOE, 2023.02; Chemical Computing Group Inc., Montreal, QC, Canada) [54] assess the plausibility of compound–target interactions by predicting binding poses and affinities. Favorable binding energies and plausible binding modes provide structural support for hypothesized interactions.

**Figure 2:**
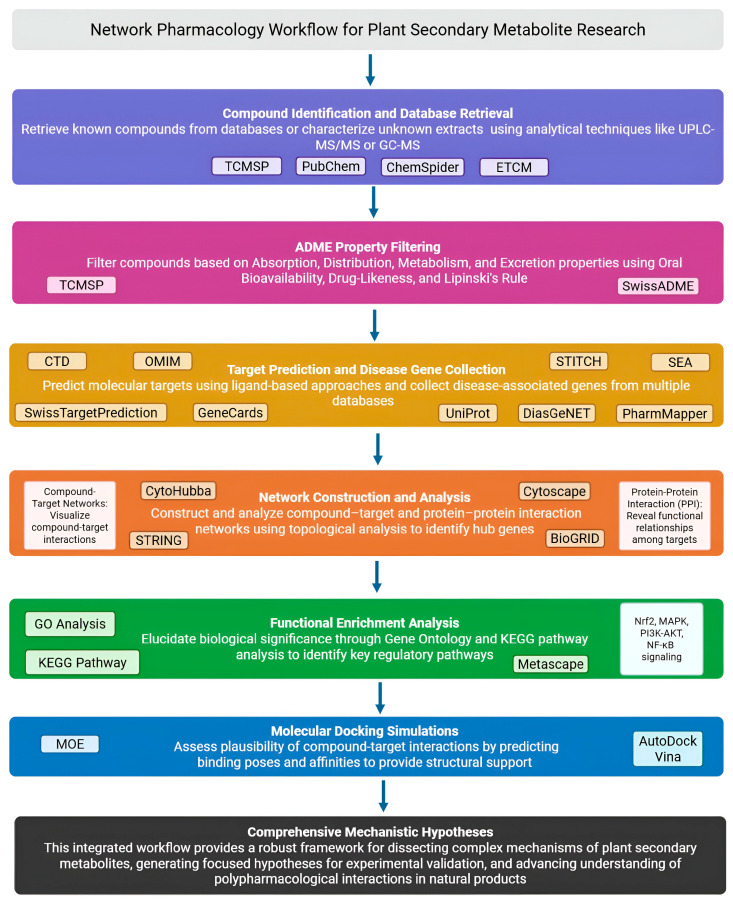
A schematic presentation of the NP workflow (Created in Biorender. Anna Merecz-Sadowska. (2025) https://BioRender.com (accessed on 15 May 2025)).

This comprehensive workflow (Figure 2), integrating computational predictions across multiple biological levels, provides a robust framework for dissecting the complex mechanisms of plant secondary metabolites and generating focused hypotheses for experimental validation [17,24].

## 4. Applying NP to Investigate the Antioxidant Potential of Secondary Metabolites

### 4.1. Introduction to the Application of NP in Antioxidant Research

Oxidative stress and corresponding antioxidant defense systems involve a complex network of biochemical reactions, signaling pathways, and regulatory elements. This includes the generation and scavenging of reactive oxygen species (ROS) and reactive nitrogen species (RNS) [55,56]; the modulation of endogenous antioxidant enzymes such as SOD, CAT, GPx, glutathione reductase (GSR), glutathione S-transferases (GST), and heme oxygenase-1 (HO-1) [57]; the activation of key transcription factors like Nrf2 [58,59]; and intricate crosstalk between redox balance and pivotal signaling cascades such as PI3K/AKT [60], MAPK [61,62], and the NF-κB pathway [63]. NP provides a holistic framework for systematically investigating how multiple components within plant extracts or classes of secondary metabolites collectively interact with this complex biological network.

This chapter provides a systematic review of studies employing NP approaches to investigate the antioxidant potential of plant secondary metabolites. It focuses on key findings regarding identified antioxidant mechanisms, molecular targets, and relevant pathways, with particular emphasis on studies combining in silico predictions with experimental validation to strengthen their conclusions.

### 4.2. Main Antioxidant Mechanisms Identified Using NP—A Review of Studies

NP, combined with experimental validation, has proven instrumental in deciphering the mechanisms underlying the antioxidant potential of plant secondary metabolites. The reviewed studies demonstrate NP’s capability to advance beyond identifying active compounds toward predicting their molecular mechanisms of action. Supplementary Table S1 presents a comparative summary of key studies utilizing NP to investigate antioxidant properties. Analysis of these studies reveals several recurring themes and molecular pathways that are frequently implicated in the antioxidant actions of natural products.

#### 4.2.1. Targeting the Nrf2/KEAP1/ARE Pathway

One of the most consistently identified mechanisms in the reviewed literature is the modulation of the Nrf2/KEAP1/ARE pathway, a central regulator of cellular antioxidant defense systems [59,64]. Under basal conditions, the transcription factor Nrf2 (encoded by NFE2L2) is sequestered in the cytoplasm by Kelch-like ECH-associated protein 1 (KEAP1) and targeted for degradation. Upon exposure to oxidative or electrophilic stress, Nrf2 dissociates from KEAP1, translocates to the nucleus, binds to Antioxidant Response Elements (AREs) in the promoter regions of target genes, and induces expression of a battery of cytoprotective enzymes, including HO-1, NAD(P)H quinone dehydrogenase 1 (NQO1), glutamate-cysteine ligase catalytic subunit (GCLC), and various glutathione S-transferases (GSTs) [65].

**Figure 3:**
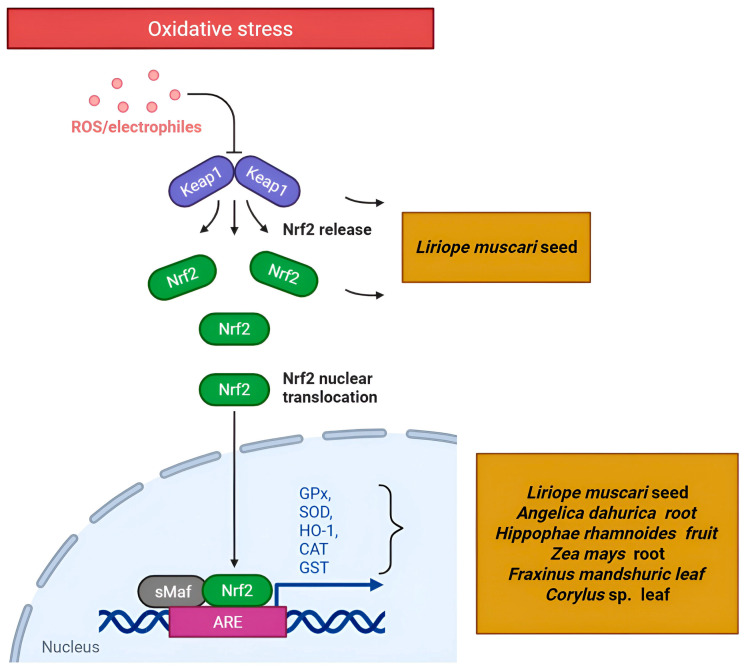
A schematic representation of the Nrf2 pathway targeted by plant secondary metabolites (Created in Biorender. Anna Merecz-Sadowska. (2025) https://BioRender.com (accessed on 15 May 2025)).

Multiple NP studies have predicted and validated this pathway (Figure 3). Truong et al. [66] demonstrated that phytosterols from *Liriope muscari* seed extract targeted KEAP1 and NFE2L2 through molecular docking, with in vitro validation showing increased CAT, GPx, and HO-1 expression. Studies on *Chimonanthus praecox* flower and *Solanum tuberosum* flesh extracts also identified AKT1 and MAPK3 as core targets linked to Nrf2 activation [67,68]. This convergence demonstrates NP’s ability to pinpoint the Nrf2 pathway as a central mechanism for diverse antioxidant metabolites.

#### 4.2.2. Modulation of Oxidative Stress-Related Signaling Pathways (PI3K/AKT, MAPK, NF-κB)

Beyond Nrf2, NP analyses frequently reveal the involvement of major signaling cascades that regulate cellular redox homeostasis [66,67,68,69,70,71,72,73,74].

The PI3K/AKT pathway, which is crucial for cell survival and proliferation, was identified as a key target in studies on *Hippophae rhamnoides* fruit flavonoids [69], *Bupleurum chinense* radix saikosaponins [75], *Litsea coreana* bud and leaf extract [71], *Chimonanthus praecox* flower extract [67], and *Solanum tuberosum* flesh extract [68]. Wang et al. [69] validated this computationally, showing that isorhamnetin glycoside protected HUVECs from H_2_O_2_-induced damage by activating AKT and downstream eNOS phosphorylation. Molecular docking confirmed potential interactions of key metabolites from *Solanum tuberosum* flesh extract and *Fraxinus mandshurica* leaf extract with AKT1 [68,76], supporting its role as a direct target.

MAPK pathways (ERK, JNK, p38) were implicated in the antioxidant mechanisms of components from *Liriope muscari* seed extract [66], *Litsea coreana* bud and leaf extract [71], *Chimonanthus praecox* flower extract [67], *Citrus sinensis* leaf extract [74], *Dracaena angustifolia* leaf extract [73], *Zea mays* root extract [72], *Corylus* sp. leaf extract [70], and *Fraxinus mandshuric* leaf extract [76]. These pathways are known responders to oxidative stress that regulate antioxidant gene expression [61].

The NF-κB pathway, while primarily inflammatory, exhibits significant redox crosstalk [77]. NP studies on *Liriope muscari* seed extract [66] and *Litsea coreana* bud and leaf metabolites [71] identified Nuclear Factor kappa B subunit 1 (NFKB1) and RELA proto-oncogene (RELA) as core targets, suggesting that antioxidant effects might be partly mediated through dampening redox-sensitive inflammatory signaling.

#### 4.2.3. Direct Interactions with Redox-Related Proteins and Enzymes

NP also predicts direct interactions with proteins that maintain redox balance. Li et al. [78] showed that polysaccharides from *Angelica dahurica* root extract could exert antioxidant effects. Their in silico analysis further predicted that the monosaccharide components of these polysaccharides, specifically rhamnose and arabinose, interact with glutathione reductase (GSR) and glutathione S-transferase A1 (GSTA1), key enzymes in glutathione metabolism. Studies identified Peroxisome Proliferator-Activated Receptor Gamma (PPARG) [68,72], known for its antioxidant effects through enzyme upregulation [79], and Caspase-3 (CASP3) [72,75], involved in oxidative stress-triggered apoptosis. Furthermore, targets that produce ROS or inflammatory mediators were frequently identified, including COX-2 (PTGS2) [66,68,76], NOS [66,80], and NADPH Oxidase 4 (NOX4) [81]. Molecular docking studies consistently supported these interactions with favorable binding energies [66,67,68,69,72,75,76,78,80].

### 4.3. Key Antioxidant Compound Classes Identified via NP

While NP primarily focuses on mechanism elucidation, the reviewed studies consistently highlight specific classes of secondary metabolites as key contributors to antioxidant effects.

Flavonoids emerge as the most prominent class, identified in extracts from *Dracaena angustifolia* leaf extract [73], *Zingiber officinale* leaf extract [82], *Hippophae rhamnoides* fruit extract [69], *Citrus sinensis* fruit extract [74], *Citrus* × *aurantium* pulp and seed extract [83], *Chimonanthus praecox* flower extract [67], *Fraxinus mandshurica* leaf extract [76], *Solanum tuberosum* flesh extract [68], and *Corylus* sp. leaf extract [70]. Key compounds, including rutin, quercetin, kaempferol, astragalin, hispidin, nobiletin, hesperidin, pinobanksin, and rhamnocitrin, were predicted to interact with core antioxidant targets.

Phytosterols (e.g., stigmasterol, β-sitosterol) and the triterpenoid cycloartenol (a precursor of numerous sterols) were identified as active compounds in *Liriope muscari* seed extract [66]. Terpenoids included saikosaponins from *Bupleurum chinense* root extract [75]. The antioxidant mechanisms of other compound classes were also investigated, including polysaccharides from *Angelica dahurica* root extract, where NP analysis focused on their constituent monosaccharides (fucose, rhamnose, arabinose, galactose, and glucose), and lignans (secoisolariciresinol, isolariciresinol) from *Chimonanthus praecox* flower extract. Other classes included an amino acid derivative (betaine) in *Zea mays* root [72] and phenolic acids (gallic acid, caffeic acid, chlorogenic acid) in *Corylus* sp. leaf extract [70].

This chemical diversity demonstrates NP’s broad applicability in identifying antioxidant mechanisms across various phytochemical classes, consistently pointing toward the modulation of Nrf2/ARE and interconnected signaling cascades as primary mechanisms.

### 4.4. Synthesis of Findings on Antioxidant Mechanisms and Comparison with Experimental Validation

The reviewed literature demonstrates good concordance between NP-predicted antioxidant potential and in vitro validation. Chemical assays (DPPH, ABTS, FRAP, hydroxyl radical scavenging) confirmed antioxidant activity in the studied extracts [66,67,68,70,72,73,75,76,78,80,82], while NP provided mechanistic depth by identifying responsible metabolites and their molecular targets.

Cell-based models using RAW 264.7 [66,70] or HUVEC [70] cells exposed to oxidative stressors, including Lipopolysaccharide (LPS) [66,70] or tert-Butyl hydroperoxide (TBHP) [70], frequently corroborated NP predictions. Studies predicting Nrf2/KEAP1 pathway involvement [67,82] observed increased expression of downstream antioxidant enzymes (HO-1, CAT, SOD, GPx) upon treatment [66,70,72]. Similarly, predictions of PI3K/AKT or MAPK pathway involvement [66,67,68,70,71,73,74,75] aligned with protection against ROS-induced damage [70]. NP-identified targets such as COX-2 or iNOS corresponded with reduced inflammatory mediators, connecting antioxidant and anti-inflammatory mechanisms [66].

#### 4.4.1. In Vivo Validation of Predicted Antioxidant Mechanisms

While less common than in vitro validation in the reviewed NP studies, in vivo experiments provide crucial confirmation of physiological relevance. Wei et al. [72] validated NP predictions for betaine from *Zea mays* root extract using an ethanol-induced oxidative stress mouse model, demonstrating increased levels of hepatic antioxidant enzymes (GSH-Px, SOD) and decreased lipid peroxidation. Similarly, Guo et al. [76] confirmed the antioxidant effects of *Fraxinus mandshurica* leaf flavonoids in vivo.

#### 4.4.2. The Role of Molecular Docking in Confirming Interactions

Molecular docking represents a key in silico validation step in the NP workflow, assessing the physical plausibility of compound–target interactions [84]. Across the reviewed studies, docking consistently indicated favorable binding energies between compounds and predicted targets. Visualization revealed specific molecular interactions including hydrogen bonds and hydrophobic contacts with key amino acid residues.

Notable examples include the following: astragalin binding to COX-2, TNF-α, and IL-2 [70]; lipophilic compounds, including phytosterols and cycloartenol (a precursor of numerous sterols), binding to targets such as KEAP1, COX-2, and iNOS [66]; flavonoids binding to AKT1 [68]; betaine binding to PPARG and COX-2 [72]; and lignans binding to ESR1, Epidermal Growth Factor Receptor (EGFR), and Proto-oncogene tyrosine-protein kinase Src (SRC) [67].

These molecular docking results provide crucial structural support for NP-hypothesized compound–target interactions, strengthening confidence in the proposed antioxidant mechanisms by demonstrating that key metabolites can physically interact with predicted targets.

## 5. Applying NP to Investigate the Anti-Inflammatory Potential of Secondary Metabolites

### 5.1. Introduction to the Application of NP in Anti-Inflammatory Research

Inflammation is a fundamental physiological process involving a complex network of signaling pathways, cellular mediators, and regulatory elements. Key molecular components include activation of transcription factors such as NF-κB [85], signaling through MAPKs [86,87], the Janus Activated Kinase/Signal Transducer and Activator of Transcription (JAK/STAT) pathway for cytokine signaling [88], and the PI3K/AKT pathway with its inflammatory crosstalk [89]. Pro-inflammatory cytokines such as TNF-α, IL-1β, and IL-6 [90], as well as enzymes including COX-2, iNOS, and Matrix Metalloproteinases (MMPs) [81,91], are hallmarks of the inflammatory cascade. NP provides a holistic framework to investigate how plant metabolites interact with this complex biological network.

This chapter systematically reviews studies employing NP to investigate the anti-inflammatory potential of plant secondary metabolites, focusing on identified mechanisms, molecular targets, and pathways, with emphasis on studies combining in silico predictions with experimental validation.

### 5.2. Main Anti-Inflammatory Mechanisms Identified Using NP—A Review of Studies

NP has been increasingly applied to unravel the complex anti-inflammatory mechanisms of plant secondary metabolites. Supplementary Table S2 presents a comprehensive summary of key studies employing NP to investigate anti-inflammatory potential. Analysis reveals recurring mechanistic themes and highlights several molecular pathways that are frequently implicated in the anti-inflammatory actions of natural products.

#### 5.2.1. Modulation of the NF-κB Signaling Pathway

**Figure 4:**
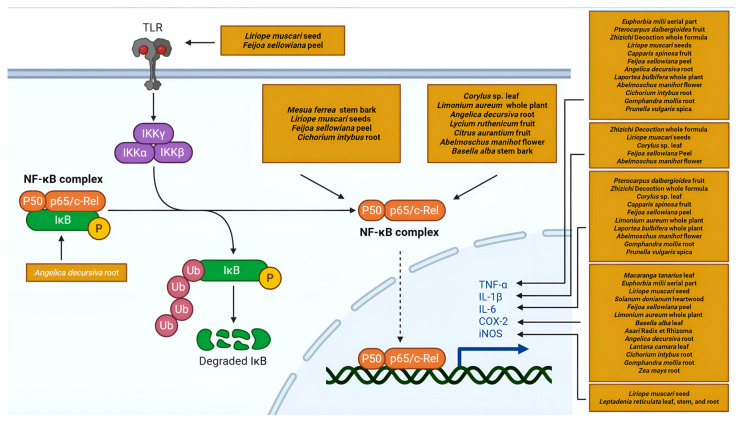
A schematic representation of the NF-κB pathway targeted by plant secondary metabolites (Created in Biorender. Anna Merecz-Sadowska. (2025) https://BioRender.com (accessed on 15 May 2025)).

The NF-κB signaling pathway stands as a pivotal regulator of inflammatory response, controlling the transcription of numerous pro-inflammatory genes. Its central role makes it a frequent target in NP analyses of anti-inflammatory natural products (Figure 4).

NP analysis of Zhizichi decoction, a traditional Chinese medicine formula composed of *Gardenia jasminoides* and *Sojae* semen praeparatum, predicted TLR4-MyD88-ERK/MAPK-NF-κB pathway involvement, with experimental validation showing reduced levels of TNF-α, IL-6, and IL-1β in LPS-induced BV2 microglia [92]. Similarly, phytosterols from *Liriope muscari* seed targeted NFKB1 through NP and molecular docking, with in vitro confirmation of reduced levels of NO, iNOS, COX-2, and IL-1β in RAW264.7 macrophages [66]. Zhao et al. demonstrated that *Citrus aurantium* Zhishi and Zhiqiao fruit extracts inhibited NF-κB signaling, suppressing p65 phosphorylation in LPS-induced RAW 264.7 cells [93].

Additional studies identify NF-κB components as crucial targets, including *Mesua ferrea* stem bark extract targeting NFKB1 [94], *Limonium aureum* whole-plant extract modulating RELA expression [95], coumarins from *Angelica decursiva* root targeting NFKBIA [96], and compounds from *Laportea bulbifera* whole plant [97] and *Gomphandra mollis* root also implicated in this pathway [98].

These studies consistently highlight NF-κB signaling as a common mechanism through which diverse plant metabolites exert anti-inflammatory effects, with modulation of NFKB1, RELA, and NFKBIA leading to the suppression of downstream inflammatory mediators.

#### 5.2.2. Involvement of MAPK Signaling Cascades

MAPK signaling pathways, including Extracellular signal-Regulated Kinase (ERK), c-Jun N-terminal Kinase (JNK), and p38 Mitogen-Activated Protein Kinase (p38 MAPK), are crucial transducers of extracellular stimuli into inflammatory responses. These pathways frequently emerge in KEGG enrichment analyses of NP studies investigating anti-inflammatory agents.

Zhizichi decoction analysis identified the TLR4-MyD88-ERK/MAPK-NF-κB pathway, with reduced pro-inflammatory cytokines supporting this prediction [92]. Zhao et al. explicitly demonstrated that *Citrus aurantium* Zhishi and Zhiqiao fruit extracts suppressed MAPK signaling in LPS-induced RAW 264.7 cells through decreased phosphorylation of MAPK/ERK Kinase Kinase 3 (MEKK3), Apoptosis Signal-regulating Kinase 1 (ASK1), and p38 MAPK [93]. Multiple studies link MAPK signaling to anti-inflammatory effects, such as *Liriope muscari* seed extract showing interactions with PRotein Kinase C Alpha/PRotein Kinase C Delta (PRKCA/PRKCD) [66], and *Limonium aureum* whole-plant extract modulating MAPK3 and JUN expression [95].

The total flavones of *Abelmoschus manihot* flower extract decreased MAPK pathway activity in Influenza A Virus-induced (IAV) lung inflammation [99]. Additional studies on compounds from *Angelica decursiva* root [96] and *Prunella vulgaris* spica [100] also implicate MAPK signaling.

These findings underscore MAPK cascades as frequent targets for plant metabolite anti-inflammatory actions, with NP successfully predicting the modulation of key proteins, with subsequent experimental confirmation.

#### 5.2.3. The Role of the PI3K/AKT Pathway in Inflammation

The PI3K/AKT pathway, while primarily known for cell survival and metabolism, exhibits significant crosstalk with inflammatory signaling and frequently appears in NP analyses of anti-inflammatory metabolites.

*Macaranga tanarius* leaf extract analysis identified PI3K/AKT signaling with prenylated flavonoids binding to AKT1 [101]. Novel compounds from *Foeniculum vulgare* fruit extract targeted AKT1 and PI3K catalytic subunit alpha, with KEGG analysis confirming PI3K/AKT involvement [102]. Similarly, *Solanum donianum* heartwood amides predicted PI3K/AKT signaling with AKT1 as a core PPI target [103].

Furthermore, a study on *Limonium aureum* whole-plant extract identified the PI3K/AKT signaling pathway as one of the major enriched KEGG pathways for anti-inflammatory targets, and experimental validation confirmed that the extract modulated AKT1 protein expression in LPS-induced macrophages [95].

AKT1 emerges as a highly recurrent hub gene across diverse plant materials, suggesting PI3K/AKT signaling modulation as a common anti-inflammatory strategy. The pathway’s regulation of cell survival and crosstalk with NF-κB and MAPK positions it as a critical therapeutic hub.

#### 5.2.4. Targeting the JAK-STAT Pathway

The JAK/STAT pathway, the primary route for transducing cytokine signals to regulate gene expression in immunity and inflammation, emerges less frequently than NF-κB, MAPK, or PI3K/AKT in NP analyses, but shows consistent importance through the key components STAT3 and JAK2. Analysis of *Feijoa sellowiana* peel extract predicted JAK2 and STAT3 as hub genes, with experimental validation showing suppressed LPS-induced phosphorylation of both proteins in RAW264.7 cells [104].

STAT3 emerged as a core target for *Capparis spinosa* fruit extract [105] and *Limonium aureum* whole-plant extract [95], suggesting JAK-STAT involvement. While *Osmanthus fragrans* flower extract targeted PI3K regulatory subunit 1 and AR for prostate cancer, these pathways exhibit crosstalk with JAK-STAT cytokine signaling [106].

The consistent prediction of JAK2 and STAT3 as molecular targets, coupled with experimental validation of their modulation, highlights this pathway’s importance in mediating the anti-inflammatory effects of diverse phytochemicals.

#### 5.2.5. Other Relevant Pathways

Beyond major signaling cascades, NP analyses reveal the involvement of additional pathways contributing to the anti-inflammatory effects of plant metabolites.

Toll-like receptor (TLR) signaling, which is crucial for innate immunity, is implicated in multiple studies: Zhizichi decoction via the TLR4-MyD88 pathway [92], *Liriope muscari* seed extract targeting TLR4 [66], and enrichment analyses for *Limonium aureum* whole-plant extract [95].

HIF-1 signaling, linking oxygen sensing to inflammation, emerged in analyses of *Macaranga tanarius* leaf extract [101]; *Alpinia oxyphylla* fruit, root, and leaf extracts [107]; *Lantana camara* leaf extract [108]; and *Leptadenia reticulata* leaf, stem, and root extracts [109].

Peroxisome Proliferator-Activated Receptor (PPAR) signaling was identified for *Citrus aurantium* Zhiqiao fruit extract, emphasizing the PPAR-AKT pathway [93], with PPARG as a hub protein in *Macaranga tanarius* leaf extract [101], and pathway modulation by *Asarum rhizome extract* [110] and *Leptadenia reticulata* leaf, stem, and root extracts [109].

Specific pathways emerge in individual studies, including Fc epsilon Receptor I (type 1) signaling (FcεRI) for *Psacalium root* cacalolides in allergic inflammation [111], cyclic Adenosine Monophosphate (cAMP) signaling for Mesua ferrea stem bark extract [94], and ovarian steroidogenesis for *Prunella vulgaris* spica extract suggesting hormonal–inflammatory intersection [100].

These findings demonstrate NP’s capacity to uncover a broader spectrum of molecular pathways, revealing common stress-response and metabolic mechanisms contributing to anti-inflammatory phenotypes across diverse plant extracts.

#### 5.2.6. Direct Interactions with Inflammatory Mediators and Enzymes

Beyond modulating signaling pathways, NP and molecular docking studies predict direct interactions between phytochemicals and key inflammatory proteins.

Cytokines, including TNF-α, IL-6, and IL-1β, are central inflammatory mediators that frequently emerge as hub genes in PPI networks. Studies identified TNF-α and IL-6 as main targets for *Mesua ferrea* stem bark extract [94]; *Pterocarpus dalbergioides* fruit extract, with docking confirmation [112]; and Zhizichi decoction, with validated cytokine reduction [92]. Additional validation came from *Liriope muscari* seed extract [66] and *Limonium aureum* whole-plant extract [95], all showing modulated cytokine expression or secretion.

Inflammatory enzymes, including COX-2, iNOS, and MMPs, that are responsible for synthesizing inflammatory mediators are also common predicted targets. COX-2, a key enzyme in prostaglandin synthesis, was consistently identified as a target for *Macaranga tanarius* leaf, with docking validation [101], and its expression was shown to be reduced by *Citrus aurantium* Zhishi and Zhiqiao fruit extracts [93], *Liriope muscari* seed extract [66], *Solanum donianum* heartwood extract [113], *Limonium aureum* whole-plant extract [95], *Basella alba* leaf extract [114], and *Angelica decursiva* root extract [96]. iNOS reduction was demonstrated for *Liriope muscari* seed extract [66] and *Euphorbia milii* aerial part extract [115], while MMP9 was targeted by *Basella alba* leaf extract [114] and *Gomphandra mollis* root extract [98].

Molecular docking consistently showed favorable binding energies between phytochemicals and these targets, strengthening the hypothesis that direct interaction with inflammatory mediators is a key mechanism for plant-derived anti-inflammatory compounds.

### 5.3. Key Anti-Inflammatory Compound Classes Identified via NP

NP studies consistently highlight specific classes of secondary metabolites as key contributors to anti-inflammatory effects, demonstrating broad applicability across diverse chemical scaffolds.

Flavonoids dominate as anti-inflammatory agents: prenylated flavonoids from *Macaranga tanarius* leaf extract targeting COX-2 [101], various flavonoids from *Citrus aurantium* Zhishi and Zhiqiao fruit extracts modulating MAPK and NF-κB [93], and compounds from *Osmanthus fragrans* flower exhibiting NO scavenging [106]. The total flavones of *Abelmoschus manihot* flower targeted TNF-α, IL-6, and MAPKs [99].

Phenolic acids feature prominently, including homogentisic acid and caffeic acid derivatives from *Euphorbia milii* aerial-part extract showing in vivo efficacy [115], and compounds from *Pterocarpus dalbergioides* fruit extract targeting TNF-α and AKT1 [112].

Terpenoids include sesquiterpenoid cacalolides inhibiting FcεRI-dependent degranulation [111].

Coumarins from *Angelica decursiva* root extract targeted TNF-α and COX-2 [96].

Lipids and fatty acid esters from *Liriope muscari* seed extract contributed to overall anti-inflammatory effects [66].

This chemical diversity demonstrates NP’s utility in identifying anti-inflammatory mechanisms across various phytochemical classes, with predicted interactions with central inflammatory pathways often supported by experimental validation.

### 5.4. Synthesis of Findings on Anti-Inflammatory Mechanisms and Comparison with Experimental Validation

The use of NP in anti-inflammatory research requires experimental validation to confirm computational predictions. The reviewed studies consistently integrate NP analyses with biological validation, following a workflow that involves the use of NP to predict active components, targets, and pathways, guiding subsequent experiments.

Overall, this iterative process of NP prediction followed by experimental validation proves fundamental for discovering and characterizing anti-inflammatory natural products.

#### 5.4.1. Concordance Between In Silico Predictions and In Vitro Anti-Inflammatory Activity

The reviewed literature demonstrates strong concordance between NP-predicted anti-inflammatory mechanisms and in vitro validation results. Cell-based models, primarily LPS-stimulated RAW264.7 macrophages [66,93,95,98,104] or BV2 microglia [66], consistently corroborate NP predictions.

Studies on Zhizichi decoction [92], *Liriope muscari* seed extract [66], *Foeniculum vulgare* fruit extract [102], *Solanum donianum* heartwood extract [103], *Feijoa sellowiana* peel extract [104], *Osmanthus fragrans* flower extract [106], and *Limonium aureum* whole-plant extract [95] demonstrated reduced NO, TNF-α, IL-6, and/or IL-1β production, aligning with NP predictions targeting pathways controlling these mediators.

Direct experimental support for the modulation of key proteins/enzymes included *Citrus aurantium* Zhishi and Zhiqiao fruit extracts modulating MAPK and NF-κB phosphorylation [93]; *Liriope muscari* seed extract reducing iNOS and COX-2 [66]; *Feijoa sellowiana* peel extract modulating JAK/STAT proteins [104]; *Limonium aureum* whole-plant extract affecting AKT1, RELA, COX-2, JUN, and MAPK3 expression [95]; and *Basella alba* leaf extract inhibiting COX-2 activity [114].

This qualitative agreement supports NP’s utility as a valuable predictive tool for the discovery of anti-inflammatory agents.

#### 5.4.2. In Vivo Validation of Predicted Anti-Inflammatory Mechanisms

In vivo studies provide critical physiological validation of NP predictions. Euphorbia milii aerial-part extract, after NP identified TNF-α, Vascular Endothelial Growth Factor A (VEGFA), PIK3CG, Epidermal Growth Factor Receptor (EGFR), and MMP9 as targets, was shown to significantly reduce carrageenan-induced paw edema in rats, with histological confirmation of decreased COX-2 and TNF-α, and modulation of pro-inflammatory (Granulocyte-Macrophage Colony-Stimulating Factor—GM-CSF, Monocyte Chemoattractant Protein 1—MCP-1, iNOS) and anti-inflammatory mediators, including IL-10 and IL-12 [115]. Similar validation approaches were employed for *Pterocarpus dalbergioides* fruit extract, where NP predicted TNF-α and AKT1 as targets, with subsequent inhibition of rat paw edema [112]. *Citrus aurantium* Zhishi and Zhiqiao fruit extract studies included LPS-induced rat models showing reduced serum IL-1β, IL-6, TNF-α, and C-Reactive Protein (CRP), with metabolomics supporting regulatory effects on inflammation-linked pathways [93].

Additional in vivo confirmations included *Asarum* rhizome extract modulating iNOS, COX-2, and Lectin-like Oxidized Low-Density Lipoprotein Receptor-1 (LOX-1) in xylene-induced ear swelling [110]; and total flavones of *Abelmoschus manihot* flower extract reducing viral load and pro-inflammatory cytokines in IAV-induced mouse lung inflammation [99].

These examples demonstrate the feasibility and importance of extending NP-guided research to whole-organism models in order to confirm the physiological relevance of predicted anti-inflammatory mechanisms.

### 5.5. The Role of Molecular Docking in Confirming Anti-Inflammatory Interactions

Molecular docking provides crucial structural validation for NP-predicted anti-inflammatory mechanisms by modeling compound–target interactions and estimating binding affinities. Across the reviewed studies, docking consistently revealed favorable binding energies between phytochemicals and inflammatory targets.

Key examples include the following: prenylated flavonoids from *Macaranga tanarius* leaf extract docking with COX-2, HSP90AA1, GSK3B, and PPARG [101]; *Mesua ferrea* stem bark compounds docking with AR, ESR1, CYP19A1, RARA, NFE2L2, TSHR, NFKB1, and ALB [94]; linoleic acid from *Pterocarpus dalbergioides* fruit [112] docking with TNF-α and AKT1; *Liriope muscari* seed phytosterols docking with COX-2, TLR4, NFKB1, KEAP1, and NOS2 [66]; *Foeniculum vulgare* fruit compounds docking with SRC, TP53, AKT1, and PIK3CA [102]; and *Solanum donianum* heartwood amides docking with COX-2 [103].

Docking poses revealed specific molecular interactions, including hydrogen bonds, hydrophobic contacts, and π-interactions with key amino acid residues. This atomistic-level validation strengthens confidence in NP-derived anti-inflammatory mechanisms, demonstrating that predicted metabolites can physically interact with identified targets.

## 6. Assessment of the Potential and Limitations of NP in Secondary Metabolite Research

### 6.1. The Emerging Prominence of NP

NP has emerged as an indispensable computational strategy for investigating plant secondary metabolites, particularly their antioxidant and anti-inflammatory properties [20,116]. This approach represents a paradigm shift from traditional single-target methodologies by providing a systems-level perspective that embraces the polypharmacological nature of natural products [17,21,117].

The core strength of NP lies in analyzing multi-target interactions within biological networks, where multiple phytochemical constituents simultaneously engage numerous molecular targets [24]. This holistic framework facilitates target identification and pathway elucidation that would be challenging to achieve through conventional reductionist approaches, providing insights into how plant extracts exert therapeutic effects through coordinated molecular interactions.

### 6.2. Therapeutic Insights and Strategic Advantages

NP is uniquely equipped to decipher the “many-to-many” relationships inherent in phytochemical action, where multiple compounds interact with diverse biological targets [21]. Across the reviewed studies, analyses consistently identified convergent molecular targets (AKT1, TNF-α, COX-2, IL-6, IL-1β, CAT, SOD, GPx) and critical signaling pathways (Nrf2, PI3K-AKT, NF-κB, MAPK), suggesting central regulatory nodes fundamental to antioxidant and anti-inflammatory responses. Furthermore, NP enabled focused hypothesis generation by predicting targets and pathways, thereby accelerating the discovery pipeline and providing mechanistic rationale for the observed bioactivities [25]. This approach also provides a framework for investigating the synergistic interactions between compounds within complex extracts [23], moving beyond isolated-constituent studies to acknowledge that therapeutic efficacy often emerges from the collective action of bioactive components.

### 6.3. Inherent Limitations and Contemporary Challenges

Despite its considerable potential, NP faces several limitations that influence result reliability and interpretability, necessitating critical evaluation of methodologies and findings.

#### 6.3.1. Database Dependency and Algorithmic Variability

NP analyses fundamentally depend on the quality, completeness, and currency of underlying databases for compounds, targets, and pathways. Inaccuracies or outdated information can propagate throughout the analytical pipeline, potentially leading to misleading conclusions [11,19]. The rapidly evolving landscape of biological data requires constant database updates, yet platforms may not incorporate the latest findings synchronously.

Furthermore, this dependency extends beyond data completeness to include significant inherent biases within curated databases. Many resources are built upon the existing literature, which has historically focused on a relatively small fraction of the proteome. This results in a “spotlight effect,” where well-studied proteins (e.g., AKT1, TNF-α, COX-2) are heavily overrepresented. Consequently, this can create a feedback loop where NP studies predominantly “rediscover” these well-established interactions, potentially overlooking novel or more specific therapeutic targets. Mitigating this bias requires a conscious effort from researchers. Key strategies to achieve this include the following: (1) cross-validating predictions using multiple platforms that employ different algorithms and underlying data sources; (2) critically assessing the biological context of top-ranked hub genes to question whether they are truly relevant or simply popular research subjects; and (3) prioritizing the experimental validation of high-scoring but less-characterized targets to break the cycle of rediscovery and foster genuine new findings.

The field employs diverse computational platforms for target prediction (TCMSP, SwissTargetPrediction, PharmMapper) and pathway analysis, utilizing different databases and algorithms. This heterogeneity can produce significant discrepancies in predicted targets and pathways for identical compound sets. Target-prediction algorithms vary from ligand-based approaches (chemical similarity, pharmacophore modeling) to structure-based methods (molecular docking), each with distinct limitations and biases (Table 1).

This variability creates a “black box” scenario where researchers often select tools based on familiarity, rather than systematic evaluation of their suitability for specific research questions or chemical spaces. Consequently, predictions should be viewed as hypotheses from specific computational pipelines rather than absolute truths, emphasizing the need for experimental validation.

#### 6.3.2. Static Models and Biological Complexity

Standard NP models provide static representations of dynamic, context-dependent biological systems. These models struggle to capture temporal dynamics, feedback loops, cell-type specificity, or differential actions of compounds as agonists versus antagonists [23]. While advanced approaches attempt to incorporate network dynamics, comprehensively modeling these complexities remains challenging.

Current workflows encounter difficulties in incorporating dose-dependencies, determining individual pharmacokinetic profiles within mixtures, or quantifying synergistic effects from complex phytochemical matrices. Most algorithms predict interactions without considering required concentrations or bioavailability at target sites. Similarly, pathway enrichment analyses identify overrepresented pathways, but do not account for pathway flux, activity levels, or specific activation contexts.

#### 6.3.3. Standardization and Methodological Transparency

The absence of universally accepted guidelines for conducting and reporting NP studies creates challenges for robust comparisons between investigations, even when examining similar extracts or compounds. Reporting inconsistencies often include insufficient detail on the database versions, algorithm parameters, or statistical thresholds applied.

This lack of standardization extends to the selection of computational tools, where researchers often choose platforms based on accessibility rather than a systematic evaluation of their suitability. While definitive recommendations are difficult given the field’s dynamic nature, guiding principles should consider the type of input material, the research objectives, and platform transparency regarding data sources and algorithms [122].

Employing complementary approaches using multiple tools can enhance prediction robustness, especially when consensus emerges. Greater methodological transparency, community-driven benchmarking efforts, and standardized reporting guidelines are crucial for enhancing rigor and reproducibility in NP studies.

Finally, our review was limited to publications in English. While this was a necessary criterion to ensure accurate interpretation and methodological consistency, we acknowledge that this approach may have introduced a language bias, potentially excluding relevant studies published in other languages. Future large-scale collaborative reviews could aim to overcome this barrier.

### 6.4. The Critical Role of Experimental Validation

The integration of NP with rigorous experimental validation is essential for translating computational predictions into biologically meaningful knowledge [20,119]. The reviewed studies demonstrate commendable concordance between NP-derived hypotheses and experimental findings, particularly regarding antioxidant and anti-inflammatory mechanisms. Frequently predicted pathways (Nrf2/ARE, PI3K/AKT, MAPK, NF-κB) and specific targets (KEAP1, AKT1, TNF-α, COX-2) have been experimentally confirmed as modulated by the studied metabolites, with molecular docking providing structural plausibility for these interactions.

While perfect predictive accuracy is not always achieved, the consistent qualitative agreement supports NP’s utility as a valuable hypothesis-generating tool. This validation process not only confirms computational predictions, but also refines our understanding of the limitations and strengths of current methodologies, creating a feedback loop that enhances future predictive accuracy.

### 6.5. Concluding Assessment

NP provides a powerful framework for navigating the complexity of plant secondary metabolites through its ability to generate testable hypotheses regarding polypharmacological mechanisms, identify key regulatory nodes, and guide experimental design. However, critical awareness of current limitations, particularly database dependencies and static model representations, remains essential for the appropriate interpretation of results.

The continued refinement of methodologies, coupled with robust experimental validation and improved standardization practices, holds significant promise for accelerating the discovery and mechanistic understanding of plant-derived compounds. The synergistic integration of computational predictions with empirical evidence is paramount for unlocking the therapeutic potential of natural products while maintaining scientific rigor and reproducibility.

## 7. Conclusions and Perspectives

Network pharmacology has established itself as a valuable and insightful approach for investigating the complex biological activities of plant secondary metabolites, particularly their antioxidant and anti-inflammatory potential. This review systematically analyzed the application of NP methodologies in this context, integrating in silico predictions with available experimental validation data. The collective findings confirm the utility of NP not only as an efficient screening tool, but also as a powerful engine for generating mechanistic hypotheses regarding how these natural compounds exert their effects at a molecular level.

The main conclusions drawn from this review are twofold. First, despite the vast chemical diversity of secondary metabolites and their varied plant origins, NP analyses reveal remarkable convergence towards a common set of molecular targets and signaling pathways underlying their antioxidant and anti-inflammatory actions. As highlighted, several key pathways, such as the Nrf2/ARE system for antioxidant defense and the NF-κB, MAPK, PI3K/AKT, and JAK/STAT signaling cascades for regulating inflammation, are consistently implicated. Core protein targets, including transcription factors (Nrf2, NFKB1, RELA, STAT3), kinases (AKT1, MAPKs, JAK2), cytokines (TNF-α, IL-6, IL-1β), and critical enzymes (KEAP1, COX-2, MMP9), repeatedly emerge as central nodes in the interaction networks. This convergence suggests that many plant metabolites achieve their protective effects by modulating these central regulatory hubs that integrate redox balance and inflammatory responses.

Second, the integration of NP with experimental validation generally shows good concordance, strengthening confidence in NP as a predictive tool. In silico predictions regarding pathway modulation and target interaction are frequently substantiated by in vitro and in vivo experiments, demonstrating the practical applicability of NP in guiding focused, hypothesis-driven research.

However, the application of NP in secondary metabolite research faces several challenges, and gaps in current knowledge need to be addressed. A significant limitation is the dependence on the quality and completeness of existing databases. Furthermore, there is a lack of standardization in NP protocols and reporting, making direct comparison between studies difficult. Few investigations explicitly compare the predictive performance of different in silico platforms or target-prediction algorithms using the same dataset. Moreover, while NP can map interactions for multiple components, effectively predicting and validating the synergistic or antagonistic effects arising from the complex interplay of numerous metabolites within an extract remains a major hurdle. Most current workflows analyze components individually before integrating targets, which may oversimplify the complex pharmacology of whole extracts.

Looking forward, several promising directions can enhance the power and reliability of network pharmacology in research on natural products. The integration of NP with other omics data, such as genomics, transcriptomics, proteomics, and metabolomics, obtained from the biological system under study offers a path towards more comprehensive and context-specific network models. This multi-omics approach can provide a more dynamic and accurate picture of cellular responses to natural-product interventions. Concurrently, the incorporation of artificial intelligence and machine learning techniques holds significant potential for improving target prediction accuracy, analyzing complex network topologies, predicting synergistic effects, and potentially automating parts of the NP workflow.

Future research should also emphasize tighter coupling between computational prediction and experimental design. NP should be used proactively to design validation experiments that are specifically aimed at testing the generated hypotheses (e.g., using specific inhibitors or genetic knockouts for predicted targets in cellular models). Addressing the limitations of current databases through improved curation and data sharing initiatives is also crucial. Finally, while this review focused on antioxidant and anti-inflammatory activities, the NP approach can and should be applied to explore the full spectrum of therapeutic potential (e.g., anticancer, neuroprotective, metabolic regulation) suggested by the complex target profiles of plant secondary metabolites.

In conclusion, network pharmacology provides an invaluable systems-level framework for deciphering the intricate mechanisms of action of plant secondary metabolites. Its ability to navigate the complexity of natural products and multifactorial biological processes positions it as a cornerstone for future research in phytotherapy and natural drug discovery. While acknowledging its current limitations, the continued development of NP methodologies, particularly through integration with multi-omics data and artificial intelligence, coupled with rigorous experimental validation, promises to significantly accelerate our understanding and exploitation of the therapeutic potential harbored within the plant kingdom.

## Figures and Tables

**Table 1:** Comparison of selected platforms and tools for target prediction and analysis in natural-product NP studies.

Feature	TCMSP	SwissTargetPrediction	PharmMapper	STITCH	ChEMBL
**Access Type**	Web-based	Web-based	Web-based	Web-based	Web-based, API
**Primary Data Source (Compounds)**	Proprietary database (mainly TCM), PubChem, ChEMBL	ChEMBL	ZINC, DrugBank, ChEMBL, TCMSP, and others	PubChem, ChEMBL	Own curated database (bioactive drug-like molecules)
**Primary Data Source (Targets/Interactions)**	DrugBank, HIT, TTD, PharmGKB, UniProt	ChEMBL (known interactions)	PDB (protein structures), DrugBank, UniProt	Experimental, predicted, database-derived, text-mined data	Literature-derived data, bioactivity data deposition
**Core Target Prediction Method**	ADME-based compound filtering (e.g., OB, DL) combined with structural similarity and pharmacokinetic properties	2D and 3D similarity (shape) to known ligands	Pharmacophore-based reverse docking	Combination of experimental, predicted (e.g., structural similarity), text mining, and homology transfer	Stores known interactions; not a primary prediction tool
**Required User Input**	Compound/TCM ingredient name, structure (SMILES)	2D/3D structure (SMILES, draw interface)	3D compound structure (mol2 format)	Compound name, SMILES, InChIKey	Compound name, structure, database ID
**Key Outputs**	Potential targets, ADME parameters, related diseases, network analysis	List of potential targets with probability scores	Ranking of potential targets (proteins) with pharmacophore fit scores	Compound–protein and protein–protein interaction networks, interaction confidence scores	Bioactivity data, targets, physicochemical properties
**Pathway/GO Analysis Integration**	Yes (e.g., KEGG, GO via linked targets)	Limited directly, targets can be exported	Limited directly, targets can be exported	Yes (e.g., KEGG, GO for associated proteins)	Data can be exported to external tools
**Special Features/Strengths**	Focus on TCM, ADME integration, user-friendly for TCM researchers	Rapid prediction for a wide range of compounds, clear interface	Identifies targets for novel compounds based on binding pocket fit	Broad scope of interactions (not just direct), confidence scoring, network visualization	Comprehensive, curated bioactivity database, standard for chemical and biological data
**Limitations/Challenges**	Limited database coverage beyond TCM, “black box” nature of some ADME parameters	Dependency on ChEMBL data quality, may favor well-studied targets	Requires 3D structure, results depend on pharmacophore model quality and coverage	Output can be very extensive, distinguishing direct vs. indirect interactions	Primarily a database rather than a prediction tool; requires API knowledge or external analytical tools for target prediction
**Typical Use in Natural-Product NP**	Identifying active ingredients and mechanisms from TCM extracts	Quick prediction of potential targets for single compounds or small libraries	Predicting targets for compounds with known 3D structures, novel target fishing	Building interaction networks, identifying key proteins in compound-related networks	Source of known activities and targets for compounds, validating predictions
**References**	[19,26]	[34,118]	[36,119]	[113]	[120,121]

## Data Availability

Not applicable.

## Supplementary Materials

The following supporting information can be downloaded at https://www.mdpi.com/article/10.3390/ijms26146678/s1.

## References

[1] Elshafie H.S., Camele I., Mohamed A.A. (2023). A Comprehensive Review on the Biological, Agricultural and Pharmaceutical Properties of Secondary Metabolites Based-Plant Origin. Int. J. Mol. Sci..

[2] Twaij B.M., Hasan M.N. (2022). Bioactive Secondary Metabolites from Plant Sources: Types, Synthesis, and Their Therapeutic Uses. Int. J. Plant Biol..

[3] Sezer F., Deniz S., Sevim D., Chaachouay N., Zidane L. (2024). Plant-Derived Natural Products: A Source for Drug Discovery and Development. Drugs Drug Candidates.

[4] Reddy V.P. (2023). Oxidative Stress in Health and Disease. Biomedicines.

[5] Pizzino G., Irrera N., Cucinotta M., Pallio G., Mannino F., Arcoraci V., Squadrito F., Altavilla D., Bitto A. (2017). Oxidative Stress: Harms and Benefits for Human Health. Oxid. Med. Cell Longev..

[6] Chen L., Deng H., Cui H., Fang J., Zuo Z., Deng J., Li Y., Wang X., Zhao L. (2017). Inflammatory Responses and Inflammation-Associated Diseases in Organs. Oncotarget.

[7] Furman D., Campisi J., Verdin E., Carrera-Bastos P., Targ S., Franceschi C., Ferrucci L., Gilroy D.W., Fasano A., Miller G.W. (2019). Chronic Inflammation in the Etiology of Disease across the Life Span. Nat. Med..

[8] Mahmoud A.M., Wilkinson F.L., Sandhu M.A., Lightfoot A.P. (2021). The Interplay of Oxidative Stress and Inflammation: Mechanistic Insights and Therapeutic Potential of Antioxidants. Oxid. Med. Cell Longev..

[9] Sasidharan S., Chen Y., Saravanan D., Sundram K.M., Yoga Latha L. (2010). Extraction, Isolation and Characterization of Bioactive Compounds from Plants’ Extracts. Afr. J. Tradit. Complement. Altern. Med..

[10] Press N.J., Joly E., Ertl P. (2019). Natural Product Drug Delivery: A Special Challenge?. Prog. Med. Chem..

[11] Simoben C.V., Babiaka S.B., Moumbock A.F.A., Namba-Nzanguim C.T., Eni D.B., Medina-Franco J.L., Günther S., Ntie-Kang F., Sippl W. (2023). Challenges in Natural Product-Based Drug Discovery Assisted with in Silico-Based Methods. RSC Adv..

[12] Romano J.D., Tatonetti N.P. (2019). Informatics and Computational Methods in Natural Product Drug Discovery: A Review and Perspectives. Front. Genet..

[13] Tessema F.B., Asfaw T.B., Tadesse M.G., Gonfa Y.H., Bachheti R.K. (2025). In Silico Studies as Support for Natural Products Research. Medinformatics.

[14] Medina-Franco J.L. (2021). Computational Approaches for the Discovery and Development of Pharmacologically Active Natural Products. Biomolecules.

[15] Liu Y., Zhang S., Liu K., Hu X., Gu X. (2024). Advances in Drug Discovery Based on Network Pharmacology and Omics Technology. Curr. Pharm. Anal..

[16] Charvériat M., Lafon V., Mouthon F., Zimmer L. (2021). Innovative Approaches in CNS Drug Discovery. Therapies.

[17] Hopkins A.L. (2008). Network Pharmacology: The next Paradigm in Drug Discovery. Nat. Chem. Biol..

[18] Sharma B., Yadav D.K. (2022). Metabolomics and Network Pharmacology in the Exploration of the Multi-Targeted Therapeutic Approach of Traditional Medicinal Plants. Plants.

[19] Zhang R., Zhu X., Bai H., Ning K. (2019). Network Pharmacology Databases for Traditional Chinese Medicine: Review and Assessment. Front. Pharmacol..

[20] Kibble M., Saarinen N., Tang J., Wennerberg K., Mäkelä S., Aittokallio T. (2015). Network Pharmacology Applications to Map the Unexplored Target Space and Therapeutic Potential of Natural Products. Nat. Prod. Rep..

[21] Efferth T., Koch E. (2010). Complex Interactions between Phytochemicals. The Multi-Target Therapeutic Concept of Phytotherapy. Curr. Drug Targets.

[22] Li S., Zhang B. (2013). Traditional Chinese Medicine Network Pharmacology: Theory, Methodology and Application. Chin. J. Nat. Med..

[23] Berger S.I., Iyengar R. (2009). Network Analyses in Systems Pharmacology. Bioinformatics.

[24] Nogales C., Mamdouh Z.M., List M., Kiel C., Casas A.I., Schmidt H.H.H.W. (2022). Network Pharmacology: Curing Causal Mechanisms Instead of Treating Symptoms. Trends Pharmacol. Sci..

[25] Wang X., Wang Z.Y., Zheng J.H., Li S. (2021). TCM Network Pharmacology: A New Trend towards Combining Computational, Experimental and Clinical Approaches. Chin. J. Nat. Med..

[26] Ru J., Li P., Wang J., Zhou W., Li B., Huang C., Li P., Guo Z., Tao W., Yang Y. (2014). TCMSP: A Database of Systems Pharmacology for Drug Discovery from Herbal Medicines. J. Cheminform..

[27] Kim S., Chen J., Cheng T., Gindulyte A., He J., He S., Li Q., Shoemaker B.A., Thiessen P.A., Yu B. (2023). PubChem 2023 Update. Nucleic Acids Res..

[28] Pence H.E., Williams A. (2010). ChemSpider: An Online Chemical Information Resource. J. Chem. Educ..

[29] Xu H.Y., Zhang Y.Q., Liu Z.M., Chen T., Lv C.Y., Tang S.H., Zhang X.B., Zhang W., Li Z.Y., Zhou R.R. (2019). ETCM: An Encyclopaedia of Traditional Chinese Medicine. Nucleic Acids Res..

[30] Wolfender J.L., Marti G., Thomas A., Bertrand S. (2015). Current Approaches and Challenges for the Metabolite Profiling of Complex Natural Extracts. J. Chromatogr. A.

[31] Atanasov A.G., Zotchev S.B., Dirsch V.M., Orhan I.E., Banach M., Rollinger J.M., Barreca D., Weckwerth W., Bauer R., Bayer E.A. (2021). Natural Products in Drug Discovery: Advances and Opportunities. Nat. Rev. Drug Discov..

[32] Lipinski C.A., Lombardo F., Dominy B.W., Feeney P.J. (2001). Experimental and Computational Approaches to Estimate Solubility and Permeability in Drug Discovery and Development Settings. Adv. Drug. Deliv. Rev..

[33] Daina A., Michielin O., Zoete V. (2017). SwissADME: A Free Web Tool to Evaluate Pharmacokinetics, Drug-Likeness and Medicinal Chemistry Friendliness of Small Molecules. Sci. Rep..

[34] Daina A., Michielin O., Zoete V. (2019). SwissTargetPrediction: Updated Data and New Features for Efficient Prediction of Protein Targets of Small Molecules. Nucleic Acids Res..

[35] Keiser M.J., Roth B.L., Armbruster B.N., Ernsberger P., Irwin J.J., Shoichet B.K. (2007). Relating Protein Pharmacology by Ligand Chemistry. Nat. Biotechnol..

[36] Wang X., Shen Y., Wang S., Li S., Zhang W., Liu X., Lai L., Pei J., Li H. (2017). PharmMapper 2017 Update: A Web Server for Potential Drug Target Identification with a Comprehensive Target Pharmacophore Database. Nucleic Acids Res..

[37] Szklarczyk D., Santos A., Von Mering C., Jensen L.J., Bork P., Kuhn M. (2016). STITCH 5: Augmenting Protein-Chemical Interaction Networks with Tissue and Affinity Data. Nucleic Acids Res..

[38] Mendez D., Gaulton A., Bento A.P., Chambers J., De Veij M., Félix E., Magariños M.P., Mosquera J.F., Mutowo P., Nowotka M. (2019). ChEMBL: Towards Direct Deposition of Bioassay Data. Nucleic Acids Res..

[39] Bateman A., Martin M.J., Orchard S., Magrane M., Ahmad S., Alpi E., Bowler-Barnett E.H., Britto R., Bye-A-Jee H., Cukura A. (2023). UniProt: The Universal Protein Knowledgebase in 2023. Nucleic Acids Res..

[40] Stelzer G., Rosen N., Plaschkes I., Zimmerman S., Twik M., Fishilevich S., Iny Stein T., Nudel R., Lieder I., Mazor Y. (2016). The GeneCards Suite: From Gene Data Mining to Disease Genome Sequence Analyses. Curr. Protoc. Bioinform..

[41] Amberger J.S., Bocchini C.A., Schiettecatte F., Scott A.F., Hamosh A. (2015). OMIM.Org: Online Mendelian Inheritance in Man (OMIM^®^), an Online Catalog of Human Genes and Genetic Disorders. Nucleic Acids Res..

[42] Piñero J., Ramírez-Anguita J.M., Saüch-Pitarch J., Ronzano F., Centeno E., Sanz F., Furlong L.I. (2020). The DisGeNET Knowledge Platform for Disease Genomics: 2019 Update. Nucleic Acids Res..

[43] Davis A.P., Grondin C.J., Johnson R.J., Sciaky D., Wiegers J., Wiegers T.C., Mattingly C.J. (2021). Comparative Toxicogenomics Database (CTD): Update 2021. Nucleic Acids Res..

[44] Kanehisa M., Furumichi M., Sato Y., Kawashima M., Ishiguro-Watanabe M. (2023). KEGG for Taxonomy-Based Analysis of Pathways and Genomes. Nucleic Acids Res..

[45] Szklarczyk D., Kirsch R., Koutrouli M., Nastou K., Mehryary F., Hachilif R., Gable A.L., Fang T., Doncheva N.T., Pyysalo S. (2023). The STRING Database in 2023: Protein-Protein Association Networks and Functional Enrichment Analyses for Any Sequenced Genome of Interest. Nucleic Acids Res..

[46] Oughtred R., Rust J., Chang C., Breitkreutz B.J., Stark C., Willems A., Boucher L., Leung G., Kolas N., Zhang F. (2021). The BioGRID Database: A Comprehensive Biomedical Resource of Curated Protein, Genetic, and Chemical Interactions. Protein Sci..

[47] Shannon P., Markiel A., Ozier O., Baliga N.S., Wang J.T., Ramage D., Amin N., Schwikowski B., Ideker T. (2003). Cytoscape: A Software Environment for Integrated Models of Biomolecular Interaction Networks. Genome Res..

[48] Chin C.H., Chen S.H., Wu H.H., Ho C.W., Ko M.T., Lin C.Y. (2014). CytoHubba: Identifying Hub Objects and Sub-Networks from Complex Interactome. BMC Syst. Biol..

[49] Carbon S., Douglass E., Good B.M., Unni D.R., Harris N.L., Mungall C.J., Basu S., Chisholm R.L., Dodson R.J., Hartline E. (2021). The Gene Ontology Resource: Enriching a GOld Mine. Nucleic Acids Res..

[50] Kanehisa M., Goto S. (2000). KEGG: Kyoto Encyclopedia of Genes and Genomes. Nucleic Acids Res..

[51] Sherman B.T., Hao M., Qiu J., Jiao X., Baseler M.W., Lane H.C., Imamichi T., Chang W. (2022). DAVID: A Web Server for Functional Enrichment Analysis and Functional Annotation of Gene Lists (2021 Update). Nucleic Acids Res..

[52] Zhou Y., Zhou B., Pache L., Chang M., Khodabakhshi A.H., Tanaseichuk O., Benner C., Chanda S.K. (2019). Metascape Provides a Biologist-Oriented Resource for the Analysis of Systems-Level Datasets. Nat. Commun..

[53] Trott O., Olson A.J. (2010). AutoDock Vina: Improving the Speed and Accuracy of Docking with a New Scoring Function, Efficient Optimization, and Multithreading. J. Comput. Chem..

[54] Morris G.M., Ruth H., Lindstrom W., Sanner M.F., Belew R.K., Goodsell D.S., Olson A.J. (2009). Software News and Updates AutoDock4 and AutoDockTools4: Automated Docking with Selective Receptor Flexibility. J. Comput. Chem..

[55] Valko M., Leibfritz D., Moncol J., Cronin M.T.D., Mazur M., Telser J. (2007). Free Radicals and Antioxidants in Normal Physiological Functions and Human Disease. Int. J. Biochem. Cell Biol..

[56] Sies H. (2015). Oxidative Stress: A Concept in Redox Biology and Medicine. Redox. Biol..

[57] Ighodaro O.M., Akinloye O.A. (2018). First Line Defence Antioxidants-Superoxide Dismutase (SOD), Catalase (CAT) and Glutathione Peroxidase (GPX): Their Fundamental Role in the Entire Antioxidant Defence Grid. Alex. J. Med..

[58] Tonelli C., Chio I.I.C., Tuveson D.A. (2018). Transcriptional Regulation by Nrf2. Antioxid. Redox. Signal..

[59] Movafagh S., Crook S., Vo K. (2015). Regulation of Hypoxia-Inducible Factor-1a by Reactive Oxygen Species: New Developments in an Old Debate. J. Cell Biochem..

[60] Manning B.D., Toker A. (2017). AKT/PKB Signaling: Navigating the Network. Cell.

[61] Son Y., Kim S., Chung H.T., Pae H.O. (2013). Reactive Oxygen Species in the Activation of MAP Kinases. Methods Enzym..

[62] Cargnello M., Roux P.P. (2011). Activation and Function of the MAPKs and Their Substrates, the MAPK-Activated Protein Kinases. Microbiol. Mol. Biol. Rev..

[63] Morgan M.J., Liu Z.G. (2011). Crosstalk of Reactive Oxygen Species and NF-ΚB Signaling. Cell Res..

[64] Hayes J.D., Dinkova-Kostova A.T. (2014). The Nrf2 Regulatory Network Provides an Interface between Redox and Intermediary Metabolism. Trends Biochem. Sci..

[65] Loboda A., Damulewicz M., Pyza E., Jozkowicz A., Dulak J. (2016). Role of Nrf2/HO-1 System in Development, Oxidative Stress Response and Diseases: An Evolutionarily Conserved Mechanism. Cell Mol. Life Sci..

[66] Truong V.L., Bae Y.J., Rarison R.H.G., Bang J.H., Park S.Y., Jeong W.S. (2023). Anti-Inflammatory and Antioxidant Activities of Lipophilic Fraction from *Liriope platyphylla* Seeds Using Network Pharmacology, Molecular Docking, and In Vitro Experiments. Int. J. Mol. Sci..

[67] Dan Z., Xiujun W., Yanbei Z., Zihang L., Xiaotian Q., Li Q. Comprehensive Metabolomics and Network Pharmacology Reveal the Mechanism of Antioxidant Activities of Chimonanthus Praecox Chemical Components. https://ssrn.com/abstract=4924486.

[68] Xu X., Chen X., Man Q., Li W., Wang L., Liu X., Chen J., Cui J. (2025). Multi-Omics, Network Pharmacology, and Molecular Docking Provide Insights into the Genetic Basis, Bioactive, and Potential Antioxidant Mechanisms in Potato (*Solanum tuberosum* L.) Flesh. Sci. Hortic..

[69] Wang M., Zhang X., Zhang Z., Tong L., Yu S., Liu Y., Yang F. (2024). Flavonoid Compounds in *Hippophae rhamnoides* L. Protect Endothelial Cells from Oxidative Damage Through the PI3K/AKT-ENOS Pathway. Chem. Biodivers.

[70] Zhao J., Wang X., Wang Y., Lv G., Lin H., Lin Z. (2023). UPLC-MS/MS Profiling, Antioxidant and Anti-Inflammatory Activities, and Potential Health Benefits Prediction of Phenolic Compounds in Hazel Leaf. Front. Nutr..

[71] Jiang S., Zhang H., Song Y., Xiao M., Hu H., Yu S., Xie F. (2025). Metabolic Profiles and Potential Antioxidant Mechanisms of Hawk Tea. Sci. Rep..

[72] Wei X., Jiao P., Jiang Z., Wang C., Liu Q., Li Y., Liu S., Guan S., Ma Y. (2025). Study on Differential Metabolite Active Ingredients in Maize Roots Based on Network Pharmacology and Metabolomics Analysis. J. Agric. Food Chem..

[73] Guo J., Wang Z., Xiang Y., Wei Z., Zheng W., Shen P., Huang L. (2024). Network Pharmacology of *Dracaena* sp. in Guangxi and Its Related Species Leaf Secondary Metabolites Possess Antioxidant Properties. Arab. J. Chem..

[74] Yang L., Li S., Chen Y., Wang M., Yu J., Bai W., Hong L. (2024). Combined Metabolomics and Network Pharmacology Analysis Reveal the Effect of Rootstocks on Anthocyanins, Lipids, and Potential Pharmacological Ingredients of Tarroco Blood Orange (*Citrus sinensis* L. Osbeck). Plants.

[75] Wang N., Li Q. (2023). Simultaneous Extraction and Analysis of Seven Major Saikosaponins from Bupleuri Radix and the Exploration of Antioxidant Activity and Its Mechanism. Molecules.

[76] Guo J., Ho C.-T., Bai N. (2024). New Utilization of *Fraxinus mandshurica* Leaves: As a Safe and Promising Natural Antioxidant. J. Agric. Food Chem..

[77] Lingappan K. (2018). NF-ΚB in Oxidative Stress. Curr. Opin. Toxicol..

[78] Li Q., Wang T., Wang C., Ding X. (2024). Ultrasonic-Assisted DES Extraction Optimization, Characterization, Antioxidant and in Silico Study of Polysaccharides from *Angelica dahurica* Radix. Arab. J. Chem..

[79] Polvani S., Tarocchi M., Galli A. (2012). PPARγ and Oxidative Stress: Con(β) Catenating NRF2 and FOXO. PPAR Res..

[80] Wonggo D., Anwar C., Dotulong V., Reo A., Taher N., Syahputra R.A., Nurkolis F., Tallei T.E., Kim B., Tsopmo A. (2024). Subcritical Water Extraction of Mangrove Fruit Extract (*Sonneratia alba*) and Its Antioxidant Activity, Network Pharmacology, and Molecular Connectivity Studies. J. Agric. Food Res..

[81] Ricciotti E., Fitzgerald G.A. (2011). Prostaglandins and Inflammation. Arter. Thromb. Vasc. Biol..

[82] Nam D.G., Kim M., Choi A.J., Choe J.S. (2024). Health Benefits of Antioxidant Bioactive Compounds in Ginger (*Zingiber officinale*) Leaves by Network Pharmacology Analysis Combined with Experimental Validation. Antioxidants.

[83] Xiao J., Sun T., Jiang S., Xiao Z., Shan Y., Li T., Pan Z., Li Q., Fu F. (2024). Antioxidant Effects and Potential Mechanisms of Citrus Reticulata ‘Chachi’ Components: An Integrated Approach of Network Pharmacology and Metabolomics. Foods.

[84] Kitchen D.B., Decornez H., Furr J.R., Bajorath J. (2004). Docking and Scoring in Virtual Screening for Drug Discovery: Methods and Applications. Nat. Rev. Drug. Discov..

[85] Liu T., Zhang L., Joo D., Sun S.C. (2017). NF-ΚB Signaling in Inflammation. Signal Transduct. Target. Ther..

[86] Arthur J.S.C., Ley S.C. (2013). Mitogen-Activated Protein Kinases in Innate Immunity. Nat. Rev. Immunol..

[87] Sabio G., Davis R.J. (2014). TNF and MAP Kinase Signalling Pathways. Seminars in Immunology.

[88] O’Shea J.J., Schwartz D.M., Villarino A.V., Gadina M., McInnes I.B., Laurence A. (2015). The JAK-STAT Pathway: Impact on Human Disease and Therapeutic Intervention. Annu. Rev. Med..

[89] Hawkins P.T., Stephens L.R. (2015). PI3K Signalling in Inflammation. Biochim. Biophys. Acta Mol. Cell Biol. Lipids.

[90] Turner M.D., Nedjai B., Hurst T., Pennington D.J. (2014). Cytokines and Chemokines: At the Crossroads of Cell Signalling and Inflammatory Disease. Biochim. Biophys. Acta Mol. Cell Res..

[91] Cinelli M.A., Do H.T., Miley G.P., Silverman R.B. (2020). Inducible Nitric Oxide Synthase: Regulation, Structure, and Inhibition. Med. Res. Rev..

[92] Chai C., Jin B., Bi J., Cui Y., Cui X., Shan C., Yu S., Wen H. (2025). Exploring of Antidepressant Components and Mechanisms of Zhizichi Decoction: Integration of Serum Pharmacochemistry, Network Pharmacology and Anti-Inflammatory Analysis Verification. Anal. Sci. Adv..

[93] Zhao S., Liu Z., Wang M., He D., Liu L., Shu Y., Song Z., Li H., Liu Y., Lu A. (2018). Anti-Inflammatory Effects of Zhishi and Zhiqiao Revealed by Network Pharmacology Integrated with Molecular Mechanism and Metabolomics Studies. Phytomedicine.

[94] Sundaram J.K., Hanumanthappa M., Kandagalla S., Shekarappa S.B., Gollapalli P., Hani U. (2023). Evaluating the Anti-Inflammatory Potential of *Mesua ferrea* Linn. Stem Bark through Network Pharmacology Approach. J. Appl. Biol. Biotechnol..

[95] Yang Z., Man J., Liu Y., Zhang H., Wu D., Shao D., Hao B., Wang S. (2023). Study on the Alleviating Effect and Potential Mechanism of Ethanolic Extract of *Limonium aureum* (L.) Hill. on Lipopolysaccharide-Induced Inflammatory Responses in Macrophages. Int. J. Mol. Sci..

[96] Li Z., Li Q. (2024). Study on the Anti-Inflammatory Mechanism of Coumarins in *Peucedanum decursivum* Based on Spatial Metabolomics Combined with Network Pharmacology. Molecules.

[97] Feng Z., Zheng Y., Pei J., Huang L. (2024). Potential Mechanism of *Laportea bulbifera* on Treating Inflammation and Tumor via Metabolomics, Network Pharmacology and Molecular Docking. J. Biomol. Struct. Dyn..

[98] Tran Huynh Q.D., Phan T.T.T., Chu M.H., Nguyen T.V., Duong T.L.T., Hsu S.J., Wang Y.H., Pham N.T., Bui B.T.N., Nguyen D.K. Anti-Inflammatory Gomphandranosides I–VIII from the Roots of Gomphandra Molis Merr. https://ssrn.com/abstract=5153553.

[99] Gao Y., Liang Z., Lv N., Shan J., Zhou H., Zhang J., Shi L. (2022). Exploring the Total Flavones of *Abelmoschus manihot* against IAV-Induced Lung Inflammation by Network Pharmacology. BMC Complement Med. Ther..

[100] Zhang Z., Su Q., Lin Y., Xia B., Li Y., Xie J., Wu P., Liao D., Lin L. (2024). The Dynamics of Bioactive Ingredients with Anti-Inflammatory and Anti-Breast Cancer Activity During *Prunellae spica* Development. Nat. Prod. Commun..

[101] Remorosa A.G.B., Tsai P.W., De Castro-Cruz K.A., Hsueh C.C., Chen R.Y., Chen B.Y. (2024). Deciphering Characteristics of *Macaranga tanarius* Leaves Extract with Electron Shuttle-Associated Anti-Inflammatory Activity via Microbial Fuel Cells, Molecular Docking, and Network Pharmacology. Biochem. Eng. J..

[102] Dong W.M., Zhang Y.T., Wang H.M., Deng M.X., Chen Z.X., He H.P., Dong F.W. (2025). Anti-Inflammatory Activities of New Compounds Isolated from the Fruits of *Foeniculum vulgare* Mill. Chem. Biodivers.

[103] Zhao J.N., Yu S.F., Wu Z.H., Chen L., Fu R., Li Z., Qu Y.L., Huang J., Wang L.B., Piao X.M. (2024). Chemical Constituents from the Heartwood of *Solanum verbascifolium* L. and Their Anti-Inflammatory Activities Combined Network Pharmacology. Chem. Biodivers.

[104] Li S., Ou P., Zhang X., Jiao G., Chen Y., Pan Y., Wang Y., Liu Q., Wang W. (2025). Anti-Inflammatory Mechanisms of *Acca sellowiana* Peel in RAW264.7 Cells: Involvement of the JAK-STAT Signaling Pathway. ACS Food Sci. Technol..

[105] Liu X., Aimaier A., Wang W., Dong Y., Han P., He J., Mu L., Wang X., Li J. (2023). Quality Variation and Biosynthesis of Anti-Inflammatory Compounds for *Capparis spinosa* Based on the Metabolome and Transcriptome Analysis. Front. Plant. Sci..

[106] Huang S.K.H., Bueno P.R.P., Garcia P.J.B., Lee M.J., De Castro-Cruz K.A., Leron R.B., Tsai P.W. (2023). Antioxidant, Anti-Inflammatory and Antiproliferative Effects of *Osmanthus fragrans* (Thunb.) Lour. Flower Extr..

[107] Ying L., Wang D., Du G. (2021). Analysis of Bioactive Components in the Fruit, Roots, and Leaves of *Alpinia oxyphylla* by UPLC-MS/MS. Evid.-Based Complement. Altern. Med..

[108] Khairan K., Maulydia N.B., Faddillah V., Tallei T.E., Fauzi F.M., Idroes R. (2024). Uncovering Anti-Inflammatory Potential of *Lantana camara* Linn: Network Pharmacology and In Vitro Studies. Narra J..

[109] Mallepura Adinarayanaswamy Y., Padmanabhan D., Natarajan P., Palanisamy S. (2024). Metabolomic Profiling of *Leptadenia reticulata*: Unveiling Therapeutic Potential for Inflammatory Diseases through Network Pharmacology and Docking Studies. Pharmaceuticals.

[110] Zhang Y., Li S., Liang Y., Liu R., Lv X., Zhang Q., Xu H., Bi K., Li Z., Li Q. (2021). A Systematic Strategy for Uncovering Quality Marker of *Asari Radix et Rhizoma* on Alleviating Inflammation Based Chemometrics Analysis of Components. J. Chromatogr. A.

[111] Castillo-Arellano J.I., Gómez-Verjan J.C., Rojano-Vilchis N.A., Mendoza-Cruz M., Jiménez-Estrada M., López-Valdés H.E., Martínez-Coria H., Gutiérrez-Juárez R., González-Espinosa C., Reyes-Chilpa R. (2018). Chemoinformatic Analysis of Selected Cacalolides from *Psacalium decompositum* (A. Gray) H. Rob. & Brettell and *Psacalium peltatum* (Kunth) Cass. and Their Effects on FcεRI-Dependent Degranulation in Mast Cells. Molecules.

[112] Alnusaire T.S., Sabouni I.L., Khojah H., Qasim S., Al-Sanea M.M., Siddique S., Mokhtar F.A., Ahmed S.R. (2023). Integrating Chemical Profiling, In Vivo Study, and Network Pharmacology to Explore the Anti-Inflammatory Effect of *Pterocarpus dalbergioides* Fruits and Its Correlation with the Major Phytoconstituents. ACS Omega.

[113] Kuhn M., von Mering C., Campillos M., Jensen L.J., Bork P. (2008). STITCH: Interaction Networks of Chemicals and Proteins. Nucleic Acids Res..

[114] Halayal R.Y., Bagewadi Z.K., Khan T.M.Y., Shamsudeen S.M. (2025). Investigating Compounds from Basella Alba for Their Antioxidant, Anti-Inflammatory, and Anticancer Properties through in Vitro and Network Pharmacology, Molecular Simulation Approach. Green Chem. Lett. Rev..

[115] Negm W.A., Elekhnawy E., Mokhtar F.A., Binsuwaidan R., Attallah N.G.M., Mostafa S.A., Moglad E., Ibrahim S., Al-Fakhrany O.M., Eliwa D. (2024). Phytochemical Inspection and Anti-Inflammatory Potential of *Euphorbia milii* Des Moul. Integrated with Network Pharmacology Approach. Arab. J. Chem..

[116] Zhai Y., Liu L., Zhang F., Chen X., Wang H., Zhou J., Chai K., Liu J., Lei H., Lu P. (2025). Network Pharmacology: A Crucial Approach in Traditional Chinese Medicine Research. Chin. Med..

[117] Joshi C.P., Baldi A., Kumar N., Pradhan J. (2024). Harnessing Network Pharmacology in Drug Discovery: An Integrated Approach. Naunyn Schmiedebergs Arch. Pharmacol..

[118] Gfeller D., Grosdidier A., Wirth M., Daina A., Michielin O., Zoete V. (2014). SwissTargetPrediction: A Web Server for Target Prediction of Bioactive Small Molecules. Nucleic Acids Res..

[119] Liu X., Ouyang S., Yu B., Liu Y., Huang K., Gong J., Zheng S., Li Z., Li H., Jiang H. (2010). PharmMapper Server: A Web Server for Potential Drug Target Identification Using Pharmacophore Mapping Approach. Nucleic Acids Res..

[120] Gaulton A., Bellis L.J., Bento A.P., Chambers J., Davies M., Hersey A., Light Y., McGlinchey S., Michalovich D., Al-Lazikani B. (2012). ChEMBL: A Large-Scale Bioactivity Database for Drug Discovery. Nucleic Acids Res..

[121] Zdrazil B., Felix E., Hunter F., Manners E.J., Blackshaw J., Corbett S., de Veij M., Ioannidis H., Lopez D.M., Mosquera J.F. (2024). The ChEMBL Database in 2023: A Drug Discovery Platform Spanning Multiple Bioactivity Data Types and Time Periods. Nucleic Acids Res..

[122] Medina-Franco J.L., Rodríguez-Pérez J.R., Cortés-Hernández H.F., López-López E. (2024). Rethinking the “best Method” Paradigm: The Effectiveness of Hybrid and Multidisciplinary Approaches in Chemoinformatics. Artif. Intell. Life Sci..

