# Supplementary material for: Network Pharmacology as a Tool to Investigate the Antioxidant and Anti-Inflammatory Potential of Plant Secondary Metabolites—A Review and Perspectives
Source: Int J Mol Sci. 2025 Jul 11;26(14):6678. doi: 10.3390/ijms26146678 (PMC12295404; doi:10.3390/ijms26146678)
Supplement: Supplementary file 1 [file ijms-26-06678-s001.zip › ijms-3718788-supplementary.pdf]

Supplementary material

Table S1. Comparative Summary of Network Pharmacology Studies Investigating the Antioxidant Potential of Secondary Metabolites.

Reference	Plant Name	Plant material / solvent	Key Identified Metabolites (via NP)	Predicted Antioxidant Targets/Pathways (via NP)	Key Hub Genes/Targets Explicitly Linked to Antioxidant Effects	In Vitro/Vivo Antioxidant Validation Method(s) Reported	Molecular Docking Performed (Yes/No), Targets related to Antioxidant Mechanism)
[66]	*Liriope muscari* (Decne.) L.H.Bailey	seed / ethanol	20 core metabolites from 43, including: glycidyl palmitate; glycidyl oleate, 1,3,12-nonadecatriene, ethyl stearate; ethyl 9,10-epoxyoctadecanoate, fucosterol, cycloartenol, clionasterol, 9-octadecenoic acid ethyl ester, butyl Linoleate, stigmasterol, 2-oxatricyclo[4.3.1.0(3,8)]decane, glyceryl monooleate, 6,7-dimethyltetralin-1,5,8-trione, diisobutyl adipate, [1R-	10 core targets from 180, including: *PTGS2, TLR4, NFE2L2, PRKCA, NFKB1, PRKCD, KEAP1, NOS2, PTGS1, NR1I2*<br>Pathways (KEGG): AGE-RAGE signaling pathway in diabetic complications, Fluid shear and atherosclerosis, IL-17 signaling pathway, TNF signaling pathway, HIF-1 signaling pathway, Toll-like receptor	*PTGS2, TLR4, NFE2L2 (Nrf2), PRKCA, NFKB1, PRKCD, KEAP1, NOS2, PTGS1, NR1I2*	In vitro:<br>Chemical-based assays: DPPH assay, ABTS assay, superoxide anion radical scavenging, hydrogen peroxide scavenging, nitric oxide radical scavenging,	Yes<br>Ligands: 20 core metabolites Targets: Top 10 core targets

[1.alpha.,2.alpha.(E)]]-butanoic acid [2-(1-hexenyl)cyclopropyl]methylester, alpha-selinene, ethyl palmitate, 3,6-diazahomoadamantan-9-ol, 2,4-decadienal	(TLR) signaling pathway, NF-kappa B signaling pathway, chemical carcinogenesis-reactive oxygen species, NOD-like receptor (NLR) signaling pathway, MAPK signaling pathway, PI3K-Akt signaling pathway.<br><br>Pathways (GO Biological Process): negative/positive regulation of apoptotic process, protein phosphorylation, inflammatory response, response to xenobiotic stimulus/lipopolysaccharide, cellular response to reactive oxygen species		hydroxyl radical scavenging, lipid peroxidation inhibition, FRAP (ferric reducing antioxidant power), PFRAP (potassium ferricyanide reducing antioxidant power), TAC (total antioxidant capacity)<br><br>Cell-based assays (LPS-stimulated RAW 264.7 cells): inhibition of ROS formation, elevation of antioxidant

						enzyme levels CAT, HO-1	
[67]	*Chimonanthus praecox* (L.) Link (Three varieties: Plain Heart (YS), Faded Heart (YY), Red Heart (YH))	flower / methanol	2,176 secondary metabolites identified	Targets: 18 core targets from 342, including: *TP53, SRC, AKT1, PIK3R1, HSP90AA1, PIK3CA, EGFR, EP300, STAT3, HSP90AB1, ESR1, MAPK3, JUN, MAPK1, MTOR, KDR, IL6, HSPA8* Pathways(KEGG): EGFR tyrosine kinase inhibitor resistance, estrogen signaling pathway, chemical carcinogenesis-receptor activation, lipid and atherosclerosis, prostate cancer Pathways (GO): negative regulation of apoptosis, cellular response to cadmium ion, positive regulation of	*ESR1, EGFR, SRC, AKT1, MAPK3*	In vitro: DPPH assay, ABTS assay, FRAP assay	Yes. Ligands: core active components identified from the network (e.g., secoisolariciresinol, isolariciresinol, 3-methylkaempferol, 6-hydroxyluteolin, laricitrin). Targets: ESR1, EGFR, SRC, AKT1, MAPK3.

[68]	*Solanum tuberosum* L.	flesh / ethanol	6 core metabolites from 985, including β-carotene, antheraxanthin, zeaxanthin, violaxanthin, neoxanthin, and lutein	transcription, cellular response to ROS<br><br>Targets:5 core targets from153, including *AKT1, PTGS2, ESR1, PPARG, SRC*<br><br>Pathways (KEGG from 80 common targets): Human diseases (cancer, drug resistance, infectious disease, cardiovascular disease, endocrine, metabolic disease), organismal systems (endocrine, immune, nervous system, aging), signal transduction (HIF-1, PI3K-Akt signaling pathways).<br><br>Pathways (GO from 80 common targets): BP: phosphorylation, negative regulation of apoptosis, negative regulation of gene	*AKT1, PTGS2, ESR1, PPARG, SRC*	In vitro: DPPH assay, ABTS assay, total flavonoid content, total anthocyanin content, total carotenoid content, Vitamin C content	Yes.<br><br>Ligands: six core compounds Targets: AKT1, PTGS2, ESR1, PPARG, SRC.

				expression. CC: cytosol, plasma membrane, cytoplasm. MF: protein binding.			
[69]	*Hippophae rhamnoides* L.	fruit / ethanol	isorhamnetin-3-*O*-glucoside 7-*O*-rhamnoside, Rrutin, laricitrin-3-*O*-rutinoside	Targets: 101 core targets<br>Pathways (KEGG): VEGF signaling pathway, PI3K-Akt signaling pathway (highlighted), focal adhesion, Rap1 signaling pathway, MAPK signaling pathway, HIF-1 signaling pathway<br>Pathways (GO molecular function): kinase-related molecular functions (e.g., kinase binding, protein kinase activity, calcium-dependent protein kinase activity)	*NOX4, AKT, eNOS*	In vitro:(HUVEC cells with H_2O_2-induced injury): cell viability (CCK8 assay), measurement of SOD, GSH, LDH, MDA levels, intracellular ROS production (DCFH-DA assay), NO release (Griess assay), mRNA expression of NOX4	Yes.<br>Ligands: isorhamnetin-3-*O*-glucoside 7-*O*-rhamnoside, rutin, laricitrin-3-*O*-rutinoside. Target: NOX4

				(GO Biological Process) included cell migration, angiogenesis, protein phosphorylation<br>GO CC included membrane raft, vesicle lumen, receptor complex		(RT-PCR), protein expression and phosphorylation of AKT and eNOS (Western blot)	
[70]	*Corylus* sp. - specifically flat-European hybrid hazelnut	leaf / methanol	17 core metabolites from 35, including: 2,5-dihydroxybenzoic acid, hydroxybenzoic acid, gallic acid, gallocatechin, p-coumaric acid, caffeic acid, chlorogenic acid, quercetin, ellagic acid, resveratrol, quercetin-3-*O*-beta-D-glucuronide, kaempferol-3-*O*-rhamnoside, luteolin, luteolin-7-*O*-glucoside, pedalitin, quercetin-3-*O*-beta-D-glucopyranoside, myricetin	Targets: 85 targets Pathways (KEGG): pathways in cancer, lipid and atherosclerosis, AGE-RAGE signaling pathway in diabetic complications, chemical carcinogenesis – receptor activation, chemical carcinogenesis – reactive oxygen species, pathways of neurodegeneration – multiple diseases, PI3K-Akt signaling pathway, fluid shear	*JUN, AKT1, VEGFA, ESR1, HSP90AA1*	In vitro: Chemical assays: DPPH assay, FRAP assay<br>Cell-based assays (TBHP-stimulated HUVEC cells): Intracellular ROS levels (DCFH-DA), SOD activity,	No

				stress and atherosclerosis		MDA levels	
[71]	*Litsea coreana* H.Lév.<br>Green tea-type hawk tea (GHT), Traditional Chinese hawk tea (TCHT), Oolong tea-type hawk tea (OHT), Black tea-type hawk tea (BHT), and Insect-type hawk tea (IHT)	bud or leaf / water-acetonitrile-isopropyl alcohol	BHT, GHT, IHT, OHT, and TCHT metabolites were 345, 348, 341, 341, and 349, respectively<br>11 core metabolites, including: adenosine, artemisinin, astragalin, bergenin, isoquercetrin, isoquercitrin, kaempferol-3-glucuronide, kaempferol 3,7,4'-trimethyl ether, tomasin, UDP-L-rhamnose, velutin	Targets: 209 targets<br>Pathways: MAPK signaling pathway, PI3K/AKT signaling pathway, pathways in cancer, apoptosis, HIF-1 signaling pathway, TNF signaling pathway, FoxO signaling pathway, cellular senescence	25 core targets, including: *MAPK1, AKT1, PIK3R1, EGFR, NFKB1, RELA, BCL2, TNF, MTOR, PRKCA, HSPA8, CCND1, SRC, GAPDH, EZH2*	No	Yes.<br>Ligands: 11 core metabolites Targets: 10 core targets: MAPK1, AKT1, PIK3R1, EGFR, NFKB1, RELA, BCL2, TNF, MTOR, PRKCA
[72]	*Zea mays* L.	root / methanol	1 core metabolite from 1154: betaine	Targets: 8 core targets from 70, including: *PPARG, PTGS2, EGFR,*	*PPARG, PTGS2, EGFR, REN,*	In vitro: DPPH assay, hydroxyl	Yes<br>Ligand: Betaine. Targets:

				REN, CASP3, ERBB2, GSK3B, HSP90AA1<br>Pathways (KEGG, from the 70 common targets): response to oxidative stress, signaling by GPCR, cellular response to nitrogen compound, regulation of system process, response to inorganic substance, regulation of postsynaptic membrane potential	*CASP3, ERBB2*	radical scavenging assay (Fenton process), antioxidant enzyme activities (CAT, SOD), gene expression of ZmCAT and ZmSOD	PPARG, PTGS2, EGFR, REN, CASP3, ERBB2
[73]	*Dracaena angustifolia* (Medik.) Roxb.*,* *Dracaena elliptica* Thunb. & Dalm., *Dracaena cochinchinensis* (Lour.) S.C.Chen, *Dracaena*	leaf / ethanol	20 core metabolites from 2971, including: choline; 2,6,10,14,18,22,26,30-dotriacontaoctaen-1-ol; 3,7,11,15,19,23,27,31-octamethyl-, (all-e)-n,n'-dicyclohexylurea;2-mercaptobenzothiazole; dipropylene glycol dimethyl ether; n,n-diisopropylethylamine; 7-hydroxycoumarine; 2,2,6,6-tetramethyl-1-	Targets: 10 core targets from 446, including: *GAPDH, AKT1, MAPK3, VEGFA, CASP3, TNF, MAPK1, SRC, EGFR, MAPK8*<br>Pathways (KEGG): AGE-RAGE signaling pathway in diabetic complications, MAPK signaling pathway,	*GAPDH, AKT1, MAPK3, VEGFA, CASP3, TNF, MAPK1, SRC, EGFR, MAPK8*	In vitro assays: DPPH, FRAP, ABTS	Yes<br>Ligands: 20 core compounds Targets: 10 core proteins

	cambodiana Pierre ex Gagnep., *Dracaena marginata* Lam, *Yucca schidigera* Roezl ex Ortgies		piperidinol; linolenic acid ethyl ester; erucamide; l-α-palmitin; citroflex 4; trans-3-indoleacrylic acid; 2-hydroxybenzothiazole; rutin; acetylcadaverine; (3beta,24r,24'r)-fucosterol epoxide; bis(4-ethylbenzylidene)sorbitol; sphinganine; diethylpyrocarbonate	HIF-1 signaling pathway			
[74]	Tarroco Blood Orange (Citrus sinensis L. Osbeck) - fruits from four different scion-rootstock combinations (Z, ZC, H, X - 'Trifoliate orange', 'Citrange', 'Hongju',	fruits / -	cyanidin-3-*O*-glucoside, hesperidin, diosmin, quercitrin, γ-linolenic acid	HIF-1 signaling, MAPK signaling, EGFR signaling, AGE-RAGE signaling pathway	*AKT1, TP53, GAPDH, MAPKs, EGFR*	DPPH, ABTS, FRAP assays	N

	'Ziyang Xiangcheng' respectively)						
[75]	*Bupleurum chinense* DC.	root / ammonia–methanol	seven major saikosaponins	Targets: 22 targets, including: *CASP3, VEGFA, STAT3, MYC, BCL2L1,* and *IL2* Pathways (KEGG): cancer pathway, PI3K-Akt signaling pathway, Hepatitis C/B pathway, JAK-STAT signaling pathway, Epstein–Barr virus infection<br>Pathways (GO Biological Process): negative regulation of apoptosis, negative regulation of gene expression, positive regulation of gene expression, protein binding, endopeptidase activity, cysteine-type endopeptidase	*CASP3, VEGFA, STAT3, MYC, BCL2L1, and IL2*	In vitro: DPPH, ABTS, Hydroxyl radical scavenging activity	Yes<br>Ligands: the seven saikosaponins Targets: CASP3, STAT3, and VEGFA

				activity involved in the apoptosis process			
[76]	*Fraxinus mandshurica* Rupr.	leaf / ethanol	7 core metabolites from 44, including: kaempferol, quercetin, dihydroquercetin, catechin, isoquercitrin, quercitrin, rutin	Targets: 147 targets from 7 core metabolites<br>Pathways (KEGG from 147 common targets): pathways in cancer, AGE-RAGE signaling pathway in diabetic complications, TNF signaling pathway, HIF-1 signaling pathway, hepatitis B, hepatitis C, Kaposi sarcoma-associated herpesvirus infection, human T-cell leukemia virus 1 infection.<br>Pathways (GO from 147 common targets): positive regulation of gene expression, DNA-templated positive regulation of transcription, response to	*PPARG, IL6, TNF, ESR1, EGFR, TP53, MMP9*	In vitro: ABTS+ radical scavenging, DPPH radical scavenging, hydroxyl radical scavenging, FRAP assay<br>In vivo (ethanol-induced oxidative stress model in mice): measurement of GSH, GSH-Px, SOD, and MDA levels in liver tissues. (Tested for	Yes.<br>Ligands: seven core compounds Targets: AKT1, TNF, ICAM1, ESR1, TOP2, PTGS2, IL6

				xenobiotic stimulus, positive regulation of transcription from RNA polymerase II promoter, response to hypoxia.		TFE and quercetin).<br>Acute oral toxicity test of TFE in rats.	
[78]	*Angelica dahurica* (Hoffm.) Benth. & Hook.f. ex Franch. & Sav.	root / ethanol	polysaccharides composed of fucose, rhamnose, arabinose, galactose, and glucose	Targets: *MAPK8, SOD2, GSTP1, NOS2, AKT1, HMOX1, HSP90A1, GSR, CASO3, PTGS2, DUSP16, PTGS1, NOS1, GSTA3, GSTM1, MAOA, HSPA8, GSTA1, MAPK12, ABL1 G8PD*<br>Pathways (GO Biological Processes): response to oxidative stress, cellular response to chemical stress, reactive oxygen species metabolic process<br>Pathways (KEGG): pathways in cancer, metabolic pathways	*GSR, GSTA1*	In vitro assays: DPPH, ABTS, OH· assays	Yes<br>Ligands: fucose, rhamnose, arabinose, galactose<br>Targets: 22 core proteins

[80]	*Sonneratia alba* Sm.	fruit / water	3 core metabolites from 21, including: ethyl Isoallocholate; Gibb-3-ene-1,10-dicarboxylic acid,2,4a,7-trihydroxy-1-methyl-8-methylene-,1,4a-lactone,10-methyl ester,(1a,2β,4aa,4bβ,10β)-; Pregn-4-ene-3,20-dione,17,21-dihydroxy-,bis(*O*-methyloxime)	Targets: 232 genes<br>Pathways: GO Biological Process): response to oxygen-containing compound, cellular response to chemical stimulus, Pathway (KEGG): pathways in cancer	*nNOS, eNOS, iNOS*	In vitro: DPPH assay	Yes<br>Ligands: 3 core compounds<br>Targets: nNOS, eNOS, iNOS
[82]	*Zingiber officinale* Roscoe	leaf / water	quercetin 3-*O*-robinobioside-7-O-rhamnoside; quercetin 3-*O*-rutinoside-7-*O*-rhamnoside; kaempferol 3-*O*-robinobioside-7-*O*-rhamnoside; kaempferol 3-*O*-rutinoside-7-*O*-rhamnoside; quercetin 3-*O*-galactoside-7-*O*-rhamnoside; quercetin 3-*O*-glucoside-7-*O*-rhamnoside; kaempferol 3-q-glucoside; kaempferol 3-*O*-galactoside-7-*O*-rhamnoside; kaempferol	Targets: *TNF, PTGS2, IL2, XDH, NOX4, AKR1B1, ACHE, ALOX5, PDE5A, CA4, CD38, NQO2, SLC29A1, TERT, ADORA3*<br>Pathways (KEGG): allograft rejection, C-type lectin receptor signaling, human T-cell leukemia virus 1 infection, metabolic pathways<br>Pathways (GO Biological Process): Negative regulation of	*TNF, PTGS2, IL2*	In vitro: DPPH assay, ABTS assays, total polyphenol content, total flavonoid content	Yes<br>Ligand: Astragalin<br>Targets: TNF, PTGS2, IL2

			3-*O*-glucoside-7-*O*-rhamnoside	endothelial cell proliferation, positive regulation of leukocyte adhesion to arterial endothelial cells, fever generation, response to fructose, negative regulation of the apoptotic process, negative regulation of vascular wound healing			
[83]	*Citrus* × *aurantium* L.	pulp, seed / methanol	11 core metabolites from 816, including: narirutin, hesperidin, neohesperidin, rutin, diosmin, didymin, hesperetin, diosmetin, sinensetin, nobiletin, tangeretin	Targets: 10 core targets from 112, including: *GAPDH, EGFR, BCL2, PTGS2, ESR1, CASP3, MMP9, AKT1, FN1, SRC*<br>Pathways (KEGG): cancer-related pathways, PI3K-Akt pathway, ROS in chemical carcinogenesis, Ras pathway, MAPK pathway	*GAPDH, EGFR, BCL2, PTGS2, ESR1*	In vitro assays: Total Phenolic Content, DPPH assay, ABTS assay, FRAP assay	No

				Pathways (GO Biological Process): protein phosphorylation, signal transduction, apoptosis inhibition, cell proliferation promotion			

Table S2. Comparative Summary of Network Pharmacology Studies Investigating the Anti-inflammatory Potential of Secondary Metabolites.

Reference	Plant Name	Plant material / solvent	Key Identified Metabolites (via NP)	Predicted Anti-inflammatory Targets/Pathways (via NP)	Key Hub Genes/Targets Explicitly Linked to Anti-inflammatory Effects	In Vitro/Vivo Anti-inflammatory Validation Method(s) Reported	Molecular Docking Performed (Yes/No), Targets related to Anti-inflammatory Mechanism
[66]	*Liriope muscari* (Decne.) L.H.Bailey	seeds / lipophilic fraction obtained by n-hexane	20 core metabolites from 43, including: glycidyl palmitate, glycidyl oleate, 1,3,12-nonadecatriene, ethyl stearate, fucosterol,	Targets: 10 core targets from 180, including: *PTGS2, TLR4, NFE2L2, PRKCA, NFKB1, PRKCD, KEAP1,*	*COX-2, TLR4, NFE2L2, PRKCA, NFKB1, PRKCD,*	In vitro: Chemical-based assays: DPPH assay, ABTS assay, superoxide	Yes. Ligands: 20 core Targets: *COX-2, TLR4, NFE2L2,*

		fractionation of 95% ethanol extract	cycloartenol, clionasterol, stigmasterol, α-selinene	*NOS2, PTGS1, NR1I2*<br>Pathways (KEGG): AGE-RAGE signaling pathway in diabetic complications, fluid shear and atherosclerosis, IL-17 signaling pathway, TNF signaling pathway, HIF-1 signaling pathway, Toll-like receptor (TLR) signaling pathway, NF-kappa B signaling pathway, Chemical carcinogenesis-reactive oxygen species, NOD-like receptor (NLR) signaling pathway, MAPK signaling pathway, PI3K-Akt signaling pathway	*KEAP1, NOS2, PTGS1, NR1I2*	anion, hydrogen peroxide, nitric oxide, hydroxyl radicals scavenging, lipid peroxidation, FRAP, PFRAP, TAC<br>Cell-based assays:(LPS-stimulated RAW 264.7 macrophages): Intracellular ROS formation, protein expression of CAT, GPx, SOD, HO-1<br>NO production, protein expression of iNOS, COX-2, IL-1β.	*PRKCA, NFKB1, PRKCD, KEAP1, NOS2, PTGS1, NR1I2*
[70]	Flat-European	leaf / methanol	quercetin-3-*O*-beta-D-glucopyranoside,	Targets: 70 targets	*RELA, JUN, AKT1,*	In vitro: (LPS-stimulated	No

	hybrid hazel *(Corylus heterophylla* Fisch., *Corylus avellana* L.)		luteolin, quercetin, chlorogenic acid, pedalitin, quercetin-3-*O*-beta-D-glucuronide, myricetin, kaempferol-3-*O*-rhamnoside, ellagic acid, resveratrol, p-coumaric acid, gallic acid, caffeic acid, hydroxybenzoic acid, gallocatechin, 2,5-dihydroxybenzoic acid	Pathways: (KEGG): cancer, lipid and atherosclerosis, coronavirus disease, herpesvirus infection, diabetes complications, TNF signaling pathway, IL-17 signaling pathway, PI3K-Akt signaling pathway	*PIK3CA, MAPK14, SYK*	RAW 264.7 cells) Measurement of NO, IL-1β, TNF-α, IL-6; (TBHP-stimulated HUVEC cells) Measurement of ROS, MDA, SOD	
[72]	*Zea mays* L.	roots / methanol	1154 metabolites<br>betaine	Targets (for Betaine): 10 core targets from 86, including: *GSK3B, PTK2B, GABRA1, HSP90AA1, ERBB2, CASP3, REN, EGFR, PTGS2, PPARG*<br>Pathways:<br>(KEGG): cellular response to nitrogen compound, regulation of system process, response	*PPARG, PTGS2, EGFR, REN, CASP3, ERBB2*	In vitro: DPPH assay, hydroxyl radical scavenging assay, hemolysis test	Ligand: Betaine<br>Targets: PPARG, PTGS2, EGFR, REN, CASP3, ERBB2

to inorganic substance, regulation of postsynaptic membrane potential, signaling by GPCR, response to oxidative stress. (GO): G protein-coupled adenosine receptor signaling pathway, G protein-coupled purinergic receptor signaling pathway, endothelin receptor signaling pathway. (CC): GABA-A receptor complex, sodium channel complex, integrin complex

(MF): G protein-coupled adenosine receptor activity, long-chain fatty acid binding, prostaglandin receptor activity

[92]	Zhizichi Decoction (ZZCD) - Gardenia jasminoides J.Ellis & Glycine max (L.) Merr.	whole formula (herbs) / water decoction	20 core metabolites from 146, including: p-coumaric acid, kaempferol, 3-methylkaempferol, quercetin, ethyl oleate, mandenol, stigmasterol, isoimperatorin, shanzhiside, scandoside methyl ester, deacetyl asperulosidic acid, geniposide, genipin 1-β-gentiobioside, gardenoside, geniposidic acid, daidzein, daidzin, genistein, genistin, glycitin	Targets: 10 core targets from 85, including: *MAP2K1, MAPK1, PIK3CG, MAPK14, PYGL, TP53, LDHA, OAT, MAOB, HSP90AA1*. Pathways (KEGG): insulin signaling, prostate cancer, Fc epsilon RI signaling, prion diseases, PPAR signaling, arginine/proline metabolism, melanoma, VEGF signaling, Non-small cell lung cancer, glioma, T-cell receptor signaling, B-cell receptor signaling, terpenoid backbone biosynthesis, GnRH signaling, Toll-like receptor signaling, glycolysis/gluconeogenesis	*TP53, HSP90AA1, MAPK1, MAPK14, MAP2K1, LDHA*	In vitro: (BV2 microglia cells): MTT assay for cytotoxicity. Measurement of LPS-induced pro-inflammatory cytokines (TNF-α, IL-6, IL-1β) release by ELISA	Yes. Ligands: 20 core metabolites Targets: 85 targets

[93]	*Citrus aurantium* L. (Zhishi and Zhiqiao)	dried young fruit and unripe fruit / ethanol	23 core metabolites from 79, including: acacetin, apigenin, naringenin, hesperidin, diosmin, nobiletin, tangeretin, poncirin, rutin, scoparone	Targets: 343 targets<br>Pathways (KEGG from 61 identified target proteins): TNF signaling pathway, NF-kappa B signaling pathway, MAPK signaling pathway, VEGF signaling pathway, Toll-like receptor signaling pathway.<br>Pathways (GO): Enrichment in biological processes related to binding and receptor activity	*MEKK3, ASK1* (targets of Zhishi), PPARg (target of Zhiqiao), *NF-kB (p65)* (common target)	In vitro: (LPS-induced RAW 264.7 cells) cytokines: TNF-α, IL-6, IL-1β (Western blot and RT-PCR)<br>**In vivo:** (LPS-induced rat model) cytokines: TNF-α, IL-6, IL-1β, CRP; metabolomics).	Yes Ligands: 79 compounds Targets: MEKK3, ASK1 (for Zhishi), PPARg (for Zhiqiao).
[94]	*Mesua ferrea* L.	stem bark / ethanol	(-)-epicatechin, 2,4-Di-tert-butylphenol, betulinic acid	Targets: 134 targets, including: *AR, ESR1, CYP19A1, RARA, NFE2L2, TSHR, NFKB1, ALB*<br>Pathways (KEGG): thyroid hormone synthesis, cAMP signaling pathway	*AR, ESR1, CYP19A1, RARA, NFE2L2, TSHR, NFKB1, ALB*	No	Yes Ligands: (-)-epicatechin, 2,4-Di-tert-butylphenol, betulinic acid Targets: AR, ESR1, CYP19A1, RARA,

							NFE2L2, TSHR, NFKB1, ALB
[95]	*Limonium aureum* (L.) Chaz.	whole plant / ethanol	1666 methabolites	Targets: 5 core tsrgets from 255: *AKT1, MAPK3, RELA, PTGS2, JUN* Pathways (GO Biological Process): inflammatory response, signal transduction, positive regulation of transcription from RNA polymerase II promoter, response to drugs, G-protein-coupled receptor signaling pathway, negative regulation of apoptotic process<br>Pathways (KEGG): pathways in cancer, cAMP signaling pathway, HIF-1 signaling pathway,	*AKT1, MAPK3, RELA, PTGS2, JUN*	In vitro: (LPS-stimulated RAW264.7 macrophages) Measurement of pro-inflammatory cytokines (IFN-γ, IL-6, TNF-α) and anti-inflammatory cytokines (IL-4, IL-10) secretion by ELISA<br>Protein Expression Validation: Western Blotting for hub genes (*AKT1, PTGS2, RELA, JUN, MAPK3*)	No

				NF-κB signaling pathway, PI3K-Akt signaling pathway, JAK-STAT signaling pathway, B cell receptor signaling pathway, Toll-like receptor signaling pathway, MAPK signaling pathway, mTOR signaling pathway.			
[96]	*Angelica decursiva* Franch. & Sav.	root / methanol	11 core metabolites, iincluding: nodakenin, imperatorin, oxypeucedanin, decursin, scoparone, praeruptorin A and B	Targets: 6 core targets from 44, including: *TNF, PTGS2, PRKACA, HSP90AB1, RELA,* NFKBIA<br>Pathways:<br>(KEGG): chemical carcinogenesis receptor activation, Pathways in cancer, IL-17 signaling pathway, cholinergic synapse pathway, regulation	*TNF, PTGS2, PRKACA, HSP90AB1, RELA, NFKBIA*	*No*	Yes Ligand: decursin Targets: TNF, PTGS2, PRKACA

				of lipolysis in adipocytes<br>(GO): inflammatory response, positive regulation of protein kinase B signaling, positive regulation of peptidyl serine physiology<br>(CC): Plasma membrane, neuron projections, cell surface<br>(MF) Protein homodimerization activity, identical protein binding, acetylcholine binding			
[97]	*Laportea bulbifera* (Siebold & Zucc.) Wedd.	whole plant / methanol	32 core methabolites from 798, including: N-feruloyltyramine, butyl isobutyl phthalate, ellagic acid, epicatechin, cynaroside	Targets: 2 core targets from 34, including *TNF, IL6*<br>Pathways:<br>(KEGG): Toll-like receptor signaling pathway, TNF	*TNF, IL-6*	No	Yes.<br>Ligands: N-feruloyltyramine, N-feruloylagmatine, ellagic acid

				signaling pathway, Pathways in cancer, IL-17 signaling pathway, PD-L1 expression and PD-1 checkpoint pathway in cancer. (GO): biological regulation, cellular process, response to stimulus.<br>(CC): cell, cell part, organelle<br>(MF): binding, catalytic activity, molecular transducer activity			Targets: TNF, IL6
[98]	*Gomphandra mollis* Merr.	root / ethanol	gomphandranosides (I-VIII), cuneataside D, 3,5-dimethoxy-4-hydroxy-benzyl alcohol 4-*O*-β-D-glucopyranoside, (+)-isolariciresinol 3a-*O*-β-glucopyranoside, (7R,8S)-dihydrodehydrodiconifer	Targets: 5 core targets from 92, including: *IL-6, TNF-α, MMP9, PTGS2, HIF1A*<br>Pathways:<br>(KEGG): NF-κB signaling pathway, IL-17 signaling pathway, TNF	*IL-6, TNF-α, MMP9, PTGS2, HIF1A*	In vitro: (LPS-stimulated RAW264.7 macrophages) Measurement: NO inhibition assay	Yes. Ligands: 16 compounds Targets: IL-6, MMP9, HIF1A, PTGS2, TNF-α

			yl alcohol 9-*O*-β-glucopyranoside	signaling pathway (GO Biological Process): astrocyte activation, neuroinflammatory response, regulation of leukocyte adhesion, inflammatory response.<br>(GO CC): extracellular space, plasma membrane components, membrane rafts. MF: cytokine receptor binding, histone-dependent DNA binding, signaling receptor activity			
[99]	*Abelmoschus manihot* (L.) Medik.	flowers / ethanol	isoquercetin, quercetin, quercetin-3'-O-glucoside, myricetin, rutin, hyperoside	Targets: 6 core targets from 62, including: *TNF, IL10, IL-6, IL-1B, JUN, MAPK1*<br>Pathways:	*TNF-α, IL-6, IL-1β, MAPK1, JUN*	In vitro: (IAV-infected MDCK or MH-S cells): Measurements: cytopathic effect, viral RNA, cytokine mRNA -	No

(KEGG): TNF signaling pathway, MAPK signaling pathway, NF-Kappa B signaling pathway, Toll-like receptor signaling pathway, RIG-I-like receptor signaling pathway<br><br>(GO): positive regulation of NF-kappa B transcription factor activity, inflammatory response, regulation of tumor necrosis factor-mediated signaling pathway<br><br>(CC): I-kappa B/NF-kappa B complex, membrane raft, cytosol<br><br>(MF) identical protein binding, protein	IL-1β, IL-6, TNF-α; MAPK phosphorylation In vivo: (IAV-infected mice) Measurements: viral load, lung cytokine mRNA, BALF cell counts, lung histology, MAPK phosphorylation.

				heterodimerization activity, cytokine activity			
[100]	*Prunella vulgaris* L.	spica (dried) / methanol	betulinic acid, oleanolic acid, ursolic acid, salvianic acid A, caffeic acid, salviaflaside, hyperoside, isoquercitrin, rosmarinic acid	Targets:17 targets<br>Pathways:<br>(KEGG): TNF signaling pathway, NOD-like receptor signaling pathway, HIF-1 signaling pathway, Rap1 signaling pathway, MAPK signaling pathway<br>(GO): CC disassembly involved in execution phase of apoptosis, proteolysis<br>(CC): cytosol, death-inducing signaling complex. (MF): cysteine-type endopeptidase activity	*CASP7, CASP8, CASP3, NOD2, CASP1*	In vitro: (LPS-induced RAW264.7 cells) Measurements: NO and IL-6 release<br>MDA-MB-231 breast cancer cells (antiproliferative activity by MTT assay)	No

[101]	*Macaranga tanarius* Müll.Arg.	leaf / ethanol and water	4 core metabolites from 21, including: (isonymphaeol B, nymphaeol A, B, C)	Targets: 519 targets<br>Pathways:<br>Pathways (GO Biological Process for 21 bioactive compounds): peptidyl-tyrosine phosphorylation, transmembrane receptor protein tyrosine kinase, signaling pathway Pathways (KEGG for 21 bioactive compounds): EGFR tyrosine kinase inhibitor resistance	*PTGS2, HSP90AA1, GSK3B, PPARG*	No	Yes Ligands: Isonymphaeol B, Nymphaeol A, Nymphaeol B, Nymphaeol C<br>Targets: PTGS2, HSP90AA1, GSK3B, PPARG
[102]	*Foeniculum vulgare* Mill.	fruit / ethanol	1-allyl-5-hydroxy-4-methoxyphenoxy-6-*O*-(E)-but-2-enoate-β-glucopyranoside, (9S,10S,16R)-octadeca-17-en-12,14-diyne-1,9,10,16-tetraol.	Targets: *SRC, TP53, AKT1, PIK3CA*<br>Pathways (KEGG): pathways in cancer, lipid and atherosclerosis, PI3K-Akt signaling pathway	*SRC, TP53, AKT1, PIK3CA*	In vitro: Inhibition of LPS-induced nitric oxide production in RAW 264.7 macrophages	No

[103]	*Solanum donianum* Walp.	heartwood / 95% ethanol	butyl cinnamoyl(4-hydroxyphenethyl) carbamate (1), N-benzoyl-L-phenylalanino (2), N-(N-benzoyl-L-phenylalanyl)-L-phenylalanol, N-p-coumaroyl tyramine, N-trans-feruloyltyramine	Targets: *SRC, TP53, AKT1, PIK3CA*<br>Pathways (KEGG): PI3K-Akt signaling pathway, NOD-like receptor signaling pathway	*SRC, TP53, AKT1, PIK3CA, COX-2*	In vitro: Inhibition of nitric oxide release in MH-S cells	Yes Ligands: 4 core compounds Target: PTGS2
[104]	*Feijoa sellowiana* (O.Berg) O.Berg	peel / polar solvent	procyanidin B1, procyanidin B2, epicatechin, ellagic acid, quercetin-3-arabinoside, hyperoside, quercetin	Targets: *ADORA1, FFAR4, NR3C2, GPR55, HDAC5, BLM, CYSLTR2, GPBAR1, HDAC9, OPRD1, EGLN1, SERPINE1, ABCC1, STK3, YES1, ACVR1B, TLR8, HIF1A, PSMB9, LYN, CBX4, NFKB1, KLF5, SCN9A, STAT1, MAOA, RET, FPR2, SLC2A1, FFAR2, SLC1A2, ACHE, RORB, RXFP1*<br>Pathways: JAK-STAT Signaling Pathway	*JAK2, STAT3, NFKB1, TLR4, PTGS2, TNF, IL6, IL1B*	In vitro: (LPS-induced RAW264.7 cells) Measurement of NO, PGE2, TNF-α, IL-6, IL-1β, iNOS, COX-2, and JAK/STAT pathway protein expression	Yes<br>Ligands: 8 core compounds Targets: JAK2 and STAT3

[105]	*Capparis spinosa* L.	fruit / ethanol	stachydrine, linoleic acid, monoolein, oleoyl ethylamide, linoleoyl ethanolamide, astragalin, chrysoeriol, diosmetin, 3,4-dihydroxybenzaldehyde, 6-gingerol, ferulic acid, vanillic acid, cappariloside a others: 2,3,5,6-tetramethylpyrazine, β-sitosterol	Targets: 167 targets Pathways:<br>(KEGG): TNF signaling pathway, NF-kappa B signaling pathway, cytokine-cytokine receptor interaction, Toll-like receptor signaling pathway, T cell receptor signaling pathway<br>(GO): Regulation of IL-6/TNF/IL-12 production, dendrite, immune receptor activity, cytokine activity	*MAPK1, STAT3, IL-6, TNF, VEGFA, SRC, MMP9, JUN*	In vitro: (LPS-induced dendritic cells); measurement of CD40, CD86, TNF-α, IL-6, IL-12p40<br>In vivo: (TPA-induced mouse ear edema)	No
[106]	*Osmanthus fragrans* Lour.	flowers / ethanol	phillygenin, ligustroside, verbascoside, 4-hydroxyphenyl acetate, rutin	Targets: 4 core targets from 42, including: *PIK3R1, Grb2, PDGFRB, AR* Pathways (GO Biological Process): Inflammatory response, response to organic	*PIK3R1, GRB2, PDGFRB, AR*	In vitro: (LPS-stimulated RAW 264.7 cells) Measurement of NO production Cytotoxicity	Yes. Ligands: phillygenin, ligustroside, verbascoside, 4-hydroxyphenyl acetate, rutin.

				substance, blood vessel endothelial cell migration, regulation of PI3K activity.<br>Pathways (KEGG): chemokine signaling pathway, prolactin signaling pathway		assay on DU-145 prostate cancer cells	Targets: PIK3R1, Grb2, PDGFRB, AR
[108]	*Lantana camara* L.	leaves / ethanol	β-acorenol, (1R,7S,E)-7-isopropyl-4,10-dimethylenecyclodec-5-enol, germacrene B, dodecanoic acid, 3-hydroxy-, neophytadiene, 9,12,15-octadecatrienoic acid (Z,Z,Z)-, n-hexadecanoic acid, β-caryophyllene, germacrene D, phytol	Targets: 5 core targets from 36, including: *PPARG, PTGS2, EGFR, HIF1A, JAK2*<br>Pathways:<br>(KEGG): Kaposi sarcoma-associated herpes virus infection, PD-L1 expression and PD-1 checkpoint pathway in cancer, Pathway in cancer, Toxoplasmosis, T helper 17 (Th17) cell differentiation	*PPARG, PTGS2, EGFR, HIF1A, JAK2*	In vitro: protein inhibition assay, albumin denaturation assay	No

(GO): inflammatory response, positive regulation of pri-miRNA transcription from RNA polymerase II promoter, negative regulation of gene expression, signal transduction, positive regulation of cytosolic calcium ion concentration

(CC): cytoplasm, cytosol, plasma membrane, nucleoplasm, external side of plasma membrane.
(MF): oxidoreductase activity, RNA polymerase II transcription factor activity, enzyme binding, protein serine/tyrosine kinase activity, protein

				phosphatase binding			
[109]	*Leptadenia reticulata* (Retz.) Wight & Arn.	leaf, stem, and root / methanol	5 core metabolites from 113, including: kaempferol, ferulic acid, luteolin, 1-pyrenylsulfate, quercitrin	Targets: 6 core targets from 520, including: *CCR2, ICAM1, KIT, MPO, NOS2, STAT3*<br>Pathways:<br>(KEGG): acute myeloid leukemia, AGE-RAGE signaling pathway in diabetic complications, HIF-1 signaling pathway (<br>(GO): reactive oxygen species biosynthesis process, reactive oxygen species metabolic process, T cell activation, T cell extravasation.<br>(CC): external side of plasma membrane, mast cell granules,	*CCR2, ICAM1, KIT, MPO, NOS2, STAT3*	No	Yes. Ligands: 18 compounds Targets: CCR2, ICAM1, KIT, MPO, NOS2, STAT3

				immunological synapses, endocytic vesicle lumen, microbody lumen.<br><br>(MF): CCR chemokine receptor binding, chemokine receptor binding, cytokine binding, heme binding.			
[110]	Asari Radix et Rhizoma (Asarum)	rhizome / ethanol	l-asarinin, okay 2-methoxy-4-vinylphenol, safrole, (3,4,5-trimethoxytoluene also, (2S)-naringenin-7-*O*-β-d-glucopyranoside, naringenin-5,4'-di-*O*-β-d-glucopyranoside	Targets:14 targets<br><br>Pathways (KEGG/Cytoscape): PPAR, NF-kB, MAPK, AMPK, Rap, Toll-like receptor signaling pathways, various pathways related to lipid metabolism	*iNOS, COX-2, LOX-1*	*In vivo: (Xylene-induced mouse ear swelling model)*<br><br>*In vitro: (from plasma of treated mice): ELISA for inflammatory factors (iNOS, COX-2, LOX-1)*	No
[111]	*Psacalium decompositum*	root/rhizome	cacalol, cacalone, cacalol acetate, maturin acetate	Targets: 50 targets<br><br>Pathways: (Reactome): Innate immune system,	*FcεRI, PI3K-Akt, MAPK*	In vitro: IgE/Antigen-dependent degranulation in	No

	(A.Gray) H.Rob. & Brettell and *Psacalium peltatum* Cass.			VEGFA-VEGFR2 pathway, Fc epsilon receptor signaling. (KEGG): PI3K-Akt pathway, MAPK signaling pathway. (GO): Protein kinase activity		bone marrow-derived mast cells	
[112]	*Pterocarpus dalbergioides* DC.	fruit / methanol	28 compounds, including: linoleic acid, 9-octadecenoic acid, 11,12,13-trihydroxy-9-octadecenoic acid, rhamnetin-3-*O*-rhamnoside.	Targets: 10 core targets from 90, including: *TNF, IL6, EGFR, MMP9, HSP90AA1, VEGFA, TP53, AKT1, CTNNB1, CCL2*.<br>Pathways (KEGG): Neuroactive ligand-receptor interaction, lipid and atherosclerosis, fluid shear stress and atherosclerosis, TNF signaling pathway, rheumatoid arthritis.	*TNF, IL6, AKT1, CCL2*	In vivo: (carrageenan-induced rat paw edema model)<br>Measurement: Paw edema volume (resulting in % inhibition compared to control)	Yes Ligands: linoleic acid, 9-octadecenoic acid, 11,12,13-trihydroxy-9-octadecenoic acid, rhamnetin-3-O-rhamnoside<br>Targets: TNF, AKT1

[114]	*Basella alba* L.	leaf / methanol	5 core methabolits from 110, including: quercetin, betacarotene, kaempferol, linoleic acid, lutein	Targets: *MMP9, AKT1, COX-2*<br><br>Pathways (KEGG): MAPK signaling pathway, PI3K-Akt signaling pathway, NF-kB signaling pathway	*COX2, MMP9, AKT1, NF-kB*	*In vitro: COX-2 enzyme inhibition assay Protein denaturation assay*<br><br>*Cytotoxic assay against MCF-7 breast cancer cells*	Yes. Ligands: quercetin, beta-carotene. Targets: MMP9, AKT1
[115]	*Euphorbia milii* Des Moul.	aerial part / ethanol	16 compounds including: homogenentisic acid, quercitrin, kaempferol-3-*O*-alpha-L-rhamnoside, astragalin, baicalein-7-*O*-glucuronide, quercetin-3-*O*-(6''-galloyl)-β-galactopyranoside, luteolin, gamma-linolenic acid, (+/-)-taxifolin.	Targets: 127 targets<br><br>Pathways (KEGG/ShinyGO): PI3K-Akt signaling pathway, MAPK signaling pathway, Ras signaling pathway.	*TNF, VEGFA, PIK3CG, EGFR, MMP9, COX-2 and TNF-α*	In vivo: (carrageenan-induced paw edema model in rats) Measurements: Paw edema weight, histological analysis (H&E staining), immunohistochemistry (COX-2, TNF-α), qRT-PCR for gene expression of proinflammatory mediators (GM-	No

CSF, MCP-1, iNOS, IL-5) and anti-inflammatory interleukins (IL-10, IL-12)

ABL1: ABL Proto-Oncogene 1, Non-Receptor Tyrosine Kinase; ABCC1 (MRP1): ATP Binding Cassette Subfamily C Member 1; ACHE: Acetylcholinesterase; ACVR1B: Activin A Receptor Type 1B; ADORA1: Adenosine A1 Receptor; ADORA3: Adenosine A3 Receptor; AGE-RAGE: Advanced Glycation End-products - Receptor for Advanced Glycation End-products; AKT / AKT1: AKT Serine/Threonine Kinase 1 (also known as PKB - Protein Kinase B); AKR1B1: Aldo-Keto Reductase Family 1 Member B1; ALB: Albumin; ALOX5: Arachidonate 5-Lipoxygenase; AMPK: AMP-Activated Protein Kinase; AR: Androgen Receptor; ASK1 (MAP3K5): Apoptosis Signal-regulating Kinase; BCL2: B-Cell Lymphoma 2; BCL2L1: BCL2 Like 1; BLM: Bloom Syndrome RecQ Like Helicase; BRAF: B-Raf Proto-Oncogene, Serine/Threonine Kinase; CA4: Carbonic Anhydrase 4; CASP1/3/7/8: Caspase 1/3/7/8; CAT: Catalase; CBX4: Chromobox 4; CCND1: Cyclin D1; CCR2: C-C Motif Chemokine Receptor 2; CD38: CD38 Molecule (Cyclic ADP Ribose Hydrolase); CTNNB1: Catenin Beta 1; CYP19A1 (Aromatase): Cytochrome P450 Family 19 Subfamily A Member 1 (Aromatase); CYSLTR2: Cysteinyl Leukotriene Receptor 2; DUSP16: Dual Specificity Phosphatase 16; EGFR: Epidermal Growth Factor Receptor; EGLN1 (PHD2): Egl-9 Family Hypoxia Inducible Factor 1; EP300: E1A Binding Protein P300; ERBB2 (HER2): Erb-B2 Receptor Tyrosine Kinase 2; ESR1: Estrogen Receptor 1; EZH2: Enhancer Of Zeste Homolog 2; FcεRI: High affinity IgE receptor; FFAR2: Free Fatty Acid Receptor 2; FFAR4: Free Fatty Acid Receptor 4; FN1: Fibronectin 1; FPR2: Formyl Peptide Receptor 2; G6PD/G8PD: Glucose-6-Phosphate Dehydrogenase (G6PD is much more common; G8PD might be a specific variant or an error in the original source); GAPDH: Glyceraldehyde-3-Phosphate Dehydrogenase; GPBAR1 (TGR5): G Protein-Coupled Bile Acid Receptor 1; GPx: Glutathione Peroxidase; GPR55: G Protein-Coupled Receptor 55; Grb2: Growth Factor Receptor Bound Protein 2; GSR: Glutathione-Disulfide Reductase; GSK3B: Glycogen Synthase Kinase 3 Beta; GSTA1/3: Glutathione S-Transferase Alpha 1/3; GSTM1: Glutathione S-Transferase Mu 1; GSTP1: Glutathione S-Transferase Pi 1; HDAC5/9: Histone Deacetylase 5/9; HIF-1 / HIF1A: Hypoxia-Inducible Factor 1 / Hypoxia Inducible Factor 1 Subunit Alpha; HMOX1 (HO-1): Heme Oxygenase 1; HSP90A1/HSP90AA1: Heat Shock Protein 90 Alpha Family Class A Member 1; HSP90AB1: Heat Shock Protein 90 Alpha Family Class B Member 1; HSPA8: Heat Shock Protein Family A (Hsp70) Member 8; ICAM1: Intercellular Adhesion Molecule 1; IFN-γ: Interferon Gamma; IL-1β: Interleukin 1 Beta; IL-2: Interleukin 2; IL-5: Interleukin 5; IL-6: Interleukin 6; IL-10: Interleukin 10; IL-12: Interleukin 12; iNOS (NOS2): Inducible Nitric Oxide Synthase (Nitric Oxide Synthase 2); JAK2: Janus Kinase 2; JUN: Jun Proto-Oncogene, AP-1 Transcription Factor Subunit; KDR (VEGFR2): Kinase Insert Domain Receptor (Vascular Endothelial Growth Factor Receptor 2); KEAP1: Kelch-Like ECH-Associated Protein 1; KIT: KIT Proto-Oncogene, Receptor Tyrosine Kinase; KLF5: KLF Transcription Factor 5; LDHA: Lactate Dehydrogenase A; LOX-1: Lectin-Like Oxidized Low-Density Lipoprotein Receptor 1; LYN: LYN Proto-Oncogene, Src Family Tyrosine Kinase; MAOA/B: Monoamine Oxidase A/B;

MAP2K1 (MEK1): Mitogen-Activated Protein Kinase Kinase 1; MAPK / MAPKs: Mitogen-Activated Protein Kinases; MAPK1/3/8/12/14: Mitogen-Activated Protein Kinase 1/3/8/12/14 (e.g., MAPK14 is p38 alpha); MCP-1 (CCL2): Monocyte Chemoattractant Protein-1; MEKK3 (MAP3K3): Mitogen-Activated Protein Kinase Kinase Kinase 3; MMP9: Matrix Metallopeptidase 9; MPO: Myeloperoxidase; MTOR: Mechanistic Target Of Rapamycin Kinase; MYC: MYC Proto-Oncogene, bHLH Transcription Factor; NFKB1 / NF-κB: Nuclear Factor Kappa B Subunit 1 / Nuclear Factor kappa B; NFKBIA: NFKB Inhibitor Alpha; nNOS (NOS1): Neuronal Nitric Oxide Synthase (Nitric Oxide Synthase 1); eNOS (NOS3): Endothelial Nitric Oxide Synthase (Nitric Oxide Synthase 3 - often NOS2 is iNOS, NOS1 is nNOS, and NOS3 is eNOS); NOS1/2: Nitric Oxide Synthase 1/2 (NOS1 - neuronal, NOS2 - inducible); NOX4: NADPH Oxidase 4; NQO2: N-Quinone Oxidoreductase 2; NR3C2: Nuclear Receptor Subfamily 3 Group C Member 2; Nrf2 (NFE2L2): Nuclear factor erythroid 2-related factor 2; OAT: Ornithine Aminotransferase; OPRD1: Opioid Receptor Delta 1; PD-1: Programmed Cell Death Protein 1; PD-L1: Programmed Death-Ligand 1; PDE5A: Phosphodiesterase 5A; PDGFRB: Platelet Derived Growth Factor Receptor Beta; PGE2: Prostaglandin E2; PI3K: Phosphoinositide 3-Kinase; PIK3CA: Phosphoinositide-3-Kinase Catalytic Alpha Polypeptide; PIK3CG: Phosphoinositide-3-Kinase Catalytic Gamma Polypeptide; PIK3R1: Phosphoinositide-3-Kinase Regulatory Subunit 1; PPARG: Peroxisome Proliferator-Activated Receptor Gamma; PRKCA: Protein Kinase C Alpha; PRKACA: Protein Kinase CAMP-Activated Catalytic Subunit Alpha; PRKCD: Protein Kinase C Delta; PSMB9: Proteasome Subunit Beta 9; PTGS1 (COX-1): Prostaglandin-Endoperoxide Synthase 1 (Cyclooxygenase-1); PTGS2 (COX-2): Prostaglandin-Endoperoxide Synthase 2 (Cyclooxygenase-2); PYGL: Glycogen Phosphorylase L; PXR (NR1I2): Pregnane X Receptor; Rap1: Rap1 GTPase; RARA: Retinoic Acid Receptor Alpha; RELA: RELA Proto-Oncogene, NF-KB Subunit; REN: Renin; RET: Ret Proto-Oncogene; RIG-I: Retinoic Acid-Inducible Gene I; RORB: RAR Related Orphan Receptor B; RXFP1: Relaxin Family Peptide Receptor 1; SCN9A; (Nav1.7): Sodium Voltage-Gated Channel Alpha Subunit 9; SERPINE1 (PAI-1): Serpin Family E Member 1 (Plasminogen Activator Inhibitor-1); SLC1A2 (GLT1): Solute Carrier Family 1 Member 2 (Glutamate Transporter 1); SLC2A1 (GLUT1): Solute Carrier Family 2 Member 1 (Glucose Transporter 1); SLC29A1: Solute Carrier Family 29 Member 1; SOD / SOD2: Superoxide Dismutase / Superoxide Dismutase 2 (mitochondrial); SRC: SRC Proto-Oncogene, Non-Receptor Tyrosine Kinase; STAT1/3: Signal Transducer and Activator of Transcription 1/3; STK3: Serine/Threonine Kinase 3; SYK: Spleen Associated Tyrosine Kinase; TERT: Telomerase Reverse Transcriptase; TLR4/8: Toll-Like Receptor 4/8; TNF / TNF-α: Tumor Necrosis Factor / Tumor Necrosis Factor alpha; TOP2: Topoisomerase (DNA) II; TP53: Tumor Protein P53; TSHR: Thyroid Stimulating Hormone Receptor; VEGFA: Vascular Endothelial Growth Factor A; VEGFR2 (KDR): Vascular Endothelial Growth Factor Receptor 2; XDH: Xanthine Dehydrogenase; YES1: YES1 Proto-Oncogene, Src Family Tyrosine Kinase
